# The Dynamics of Supply and Demand in mRNA Translation

**DOI:** 10.1371/journal.pcbi.1002203

**Published:** 2011-10-13

**Authors:** Chris A. Brackley, M. Carmen Romano, Marco Thiel

**Affiliations:** 1 Institute for Complex Systems and Mathematical Biology, SUPA, University of Aberdeen, Aberdeen, United Kingdom; 2 Institute of Medical Sciences, Foresterhill, University of Aberdeen, Aberdeen, United Kingdom; Virginia Polytechnic Institute & State University, United States of America

## Abstract

We study the elongation stage of mRNA translation in eukaryotes and find that, in contrast to the assumptions of previous models, both the supply and the demand for tRNA resources are important for determining elongation rates. We find that increasing the initiation rate of translation can lead to the depletion of some species of aa-tRNA, which in turn can lead to slow codons and queueing. Particularly striking “competition” effects are observed in simulations of multiple species of mRNA which are reliant on the same pool of tRNA resources. These simulations are based on a recent model of elongation which we use to study the translation of mRNA sequences from the *Saccharomyces cerevisiae* genome. This model includes the dynamics of the use and recharging of amino acid tRNA complexes, and we show via Monte Carlo simulation that this has a dramatic effect on the protein production behaviour of the system.

## Author Summary

In this paper we show that the rate at which proteins are produced can be controlled at the elongation stage of mRNA translation. Regulation of translation initiation has been a focus of much study, but the subsequent effect of changes in the initiation rate on the overall translation rate, and the role of slow and fast codon usage in mRNA sequences is still not fully understood. We consider a model of elongation in which the dynamics of tRNA use and recharging are considered for real mRNA sequences. We find that the balance between the demand for, and supply of tRNAs is crucial in determining translation rates. Particularly interesting “competition” effects are observed when the simultaneous translation of multiple mRNA is considered. We show indeed that, via the choice of slow or fast codons, it is in principle possible to control how variation of the supply and demand for tRNA resources changes the rate of protein production from different mRNAs.

## Introduction

The translation of mRNAs by ribosomes is one of the steps in protein synthesis, and as such underpins all cellular processes. Control pathways at the transcriptional level are a long studied phenomenon, but it is becoming clear that control of protein production could also be exercised at the level of translation [1]–[4]. Proteins are assembled from their constituent amino acids by molecular machines called ribosomes, which move along the open reading frame (ORF) of an mRNA. Translation of the mRNA proceeds in three separate stages: initiation, where ribosomes form at the 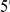 end of the mRNA, and scan along until they encounter a “start codon” (almost always AUG); elongation, where amino acids are provided to the ribosome via chaperoning transfer RNA (tRNA) molecules, and are added to the growing polypeptide; and finally termination, when a ribosome detaches from the mRNA at a stop codon, and the polypeptide chain is released ready for folding or further processing by the cellular machinery. At each stage there is opportunity for control of protein production. In this paper we consider control during elongation, employing a model which takes into account the varying rates of translation of different codons.

Ribosomes translate the ORF in a stepwise manner. Each codon (three nucleotides) codes for a specific amino acid. The ribosome waits at each codon until the correct amino acid tRNA (aa-tRNA) complex binds with its A site [5]; the amino acid is then transferred to the growing peptide chain, and the ribosome advances to the next codon. Bare tRNAs are released back into the cytoplasm, where they are reused after being “recharged” with a new amino acid. In *Saccharomyces cerevisiae* there are 41 tRNA species, each carrying a specific one of the 20 common amino acids - i.e., in general there is more than one tRNA species carrying the same amino acid. It is thought that the rate at which the ribosome translates a specific codon type depends on the abundance of the relevant aa-tRNA molecule [3], [6]. Some tRNAs are very abundant, whilst others are relatively rare; in fact there are amino acids for which there exists both an abundant tRNA and a rare tRNA. This poses the question as to what benefit there could be for the cell to sometimes use a codon which codes for a rare tRNA (which we shall call a slow codon), when a more quickly translated alternative exists. That is, what benefit is there in introducing ribosome bottlenecks or pauses to translation? The answer to this question is likely to be multifaceted, for example it may reduce the error rate and risk of premature termination. Here we consider whether bottlenecks might also be used to enact control on protein production; this could have major impact on our understanding of the role of translation both in wide type and synthetic biology applications.

In this paper we show that it is the interplay between the demand for and the supply of tRNA resources which determines the existence of bottlenecks to translation, and ultimately how this controls protein production. For example if the demand for a particular tRNA is very high, then the elongation of the corresponding codons can become the rate limiting step of translation, even if the abundance of that tRNA is high. That is to say, the availability of a species of charged aa-tRNA depends not only on the tRNA abundance, as assumed in previous works, but also on the demand for that species. We examine how translation of different mRNAs is coupled through a common pool of resources. We note that the present work is in contrast to previous studies which have considered the effect of a finite pool of ribosomes [7], which leads to very different effects on the translation dynamics.

In general several ribosomes can elongate the same mRNA at once; this can lead to the formation of queues of ribosomes, as they cannot overtake each other. Thus the occupancy by ribosomes of different parts of an mRNA gives information about the translation of that gene [8]. Elongation is often treated using traffic models, and here we apply a model where excluding “particles” take discrete steps along a one dimensional lattice; this has been detailed extensively in the non-equilibrium statistical mechanics literature [9]–[11]. This model, known as the totally asymmetric exclusion process (TASEP), has been recently extended by Brackley et al. [12] to take into account the fact the abundance of different aa-tRNA molecules can actually vary with time. Previous work [13]–[15] has assumed that all tRNAs are always bound to an amino acid. That is, they assume that aa-tRNA abundances, and therefore different codon types' translation rates, are constant. We show by relaxing this assumption, that it is not only the abundance of tRNAs which determines translation rates, but one must also consider the dynamics of both the supply of and the demand for tRNAs. Importantly, the balance between supply and demand is likely to change due to environmental influences.

In the next section we describe the model and the method by which we perform simulations. We then investigate how the rate of translation initiation affects protein production, studying several mRNA sequences from the *Saccharomyces cerevisiae* genome, and comparing with results from a model where aa-tRNA levels are fixed. We first consider each mRNA sequence separately, performing simulations with multiple copies of the same mRNA. Finally we consider different mRNA species using the same tRNA resource pool. We study how competition for different resources can change protein production rates depending on the number of each species of mRNA. We present results from simulations with two mRNA species, and larger scale simulations which contain a representative mixture of up to 70 mRNA species all in contact with the same pool of tRNAs. In the large scale simulations we consider changes to the abundance of some mRNA types on a scale which will occur during the normal life cycle of the cell, and show that this can result in a significant change in the production rate of some proteins.

## Methods

**Figure 1:**
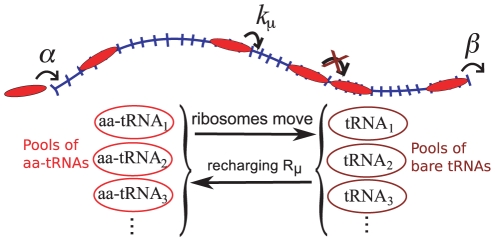
Schematic representation of the model including use and recharging of tRNAs. Red particles represent ribosomes, and the lattice represents the mRNA. Ribosomes move from site to site with rates dependent on the size of a pool of aa-tRNAs. Every time a ribosome moves out of a site of type 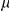, a 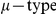 aa-tRNA is removed from the pool, and a 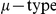 tRNA is added to the corresponding pool of bare tRNAs. Bare 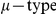 tRNAs are recharged with a rate 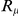.

The TASEP is a stochastic model whereby particles, here representing ribosomes, hop along a 1D lattice of sites, here representing the codons of an mRNA. The system is represented schematically in Fig. 1. Although only one codon is “read” at a time, the ribosomes actually cover several codons. They enter the ORF with a rate 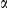 provided there is not another ribosome blocking the entry. In reality the initiation rate depends on the local nucleotide sequence (particularly in the 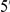 leader region, where secondary structures may form [16]) and so is mRNA specific. Here for simplicity we assume all mRNAs have the same initiation rate, and we will vary this as a control parameter in simulations. The ribosomes then hop from codon to codon in a rightward direction, as depicted in the figure, with a rate dependent on the type of codon. We label the codon positions from left to right 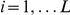, and label their species 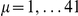. The labels for the codon species are assigned via an alphabetical list of the corresponding tRNAs; a key is available in the supporting information (Text S2) associated with this article. Once they reach the end of the lattice, the ribosomes leave with a rate 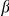. It is thought that termination is not a limiting step in translation [17], so in the remainder of this paper we assume that 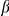 is larger than all of the other rates.

As in [12] we include the fact that when a ribosome hops from one codon to the next an aa-tRNA is used, leaving a bare tRNA. It then takes a finite time for this to be recharged with a new amino acid. Every time a ribosome hops from a codon of type 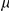, we reduce the number of 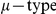 aa-tRNAs by one. We assume that the hopping rate for each codon type is linearly dependent on the number of aa-tRNAs
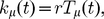
(1)where 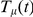 is the number of aa-tRNA molecules of type 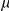 available at time 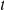. We expect that 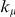 would actually saturate for large 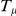, but the linear approximation is justified, since on energetic grounds the cell in unlikely to overproduce tRNAs. We assume that the total number of tRNAs (charged and uncharged) of each species 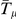 is constant, and estimate values from their gene copy numbers (see parameters section). We denote the total number of tRNAs of all types
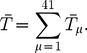
(2)


The recharging of bare tRNAs with new amino acids is an enzymatic process, facilitated by a family of synthetases. For simplicity we assume that the availability of amino acid molecules is not limiting, and model the recharging rate using a Michaelis-Menten equation [18], [19]

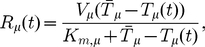
(3)where 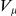 is the maximum recharging rate of the 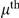 tRNA type, and 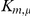 is the number of bare 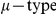 tRNAs for which the rate is half maximum. The exact form of the equation for recharging does not qualitatively affect the results.

Provided the rates 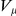 are small enough (which is the case for realistic parameters - see [12], [20]) interesting effects are seen when the rate at which aa-tRNAs are used approaches the rate at which they are recharged. For some very simple designer mRNA sequences it is possible to solve the model analytically using a mean field approach [20], but in order to treat realistic mRNA sequences we must resort to Monte Carlo simulations as discussed below.

We quote here some steady state results for a uniform mRNA with only one type of codon, which will be useful in our later analysis. For small initiation rates 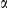 there are only a small number of ribosomes on the mRNA at any one time, and the current 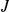 and mean density 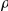 of ribosomes in this “low density” or LD phase are as follows [12], [21], [22]

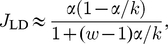
(4)

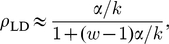
(5)where we use 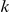 without an index to indicate that there is only one type of codon, and 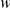 is the width of the ribosomes in units of codons. In the steady state the current 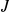 is the average rate at which ribosomes will pass any point on the mRNA, and is therefore equivalent to the protein production rate. By the density 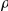 we mean the proportion of the mRNA which is covered by the “reader” part of the ribosomes; an alternative measure is the coverage density 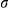 which refers to the proportion of the mRNA covered by any part of the ribosome. As 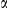 is increased both the current and density of ribosomes increase, as does therefore, the rate at which tRNAs are used. When the rate of tRNA use reaches the rate at which they are recharged the behaviour changes: the current no longer increases, and 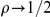. We denote this the “limited resources” or LR regime, and the initiation rate at which it is reached can be approximated
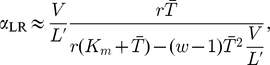
(6)where 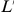 denotes the total number of codons in the system, and again we use symbols without the subscript for this case where there is only one type of codon. In this LR regime the charging level (defined as 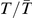) decreases, and the translation rate is reduced. We note that Refs. [12], [20] detail a model which includes elongation rates which vary with the availability of charged tRNA, but assumes ribosomes which obscure only the codon they are elongating, and Refs. [21]–[23] consider a model with fixed elongation rates, but extended ribosomes. Here we combine these models; full details are given in the supporting information (Text S1).

Introducing multiple codon types means that, in general, as 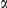 is increased only some specific tRNA use rates will approach their recharging rates. We call a regime where the charging level of 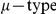 tRNAs becomes depleted a 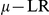 regime. Multiple codon types can also lead to the formation of queues of ribosomes. This happens if the translation rate for a codon somewhere in the bulk of the mRNA is both lower than that of the preceding codons, and is sufficiently smaller than the initiation rate [20], [23], [24]. There are two possible routes to queueing of ribosomes behind certain codons: either (a) there is a tRNA with such a low total abundance (low 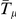) that this causes queueing before aa-tRNAs become limited, or (b) the abundance of charged aa-tRNAs becomes depleted in a 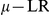 regime (low 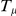), and ribosomes queue behind the corresponding codons. Case (a) is analogous to the queueing phase (QP) transition in the original TASEP model without recharging, which has been studied extensively in the literature [11], [23]–[27]. Hence we will refer to this as QP. Case (b) is qualitatively different in that there is a smooth onset of the 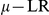 regime as the initiation rate is increased, which results in queueing. We refer to this as “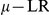 induced queueing” in order to distinguish it from the QP. We discuss these different types of behaviour in more detail in the results section.

### Monte Carlo Simulations

Simulations proceed via a similar scheme used in many previous studies of the TASEP (for example see [9]), where codon sites are chosen at random. We use continuous time Monte Carlo methods as this is very efficient [28]. If a 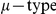 codon is being read by a ribosome, the ribosome advances with rate 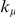 provided the next codon is vacant and there is a 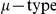 aa-tRNA available. In each simulation we treat 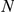 copies of a particular mRNA attached to the same pool of tRNAs. To model initiation we also include a 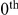 codon for each mRNA, which always contains a ribosome ready to enter the lattice with rate 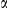. To include recharging, we not only pick from the 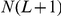 codon sites, but also from the 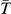 tRNAs. Unbound 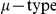 tRNAs are recharged with rate 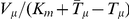 per tRNA. In order to eliminate any transient effects due to the initial conditions, we disregard the first 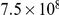 Monte Carlo steps (MCS) and run for a further 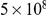 MCS; i.e., all results shown are for the steady state.

### Parameters

Throughout this paper we use parameters which match those found experimentally for the widely studied yeast *Saccharomyces cerevisiae*. A typical yeast cell contains a total of 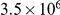 codons (based on mRNA abundances from [29]) and 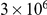 tRNAs [30]. In order to be able to efficiently perform simulations we study smaller systems, typically containing 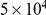 codons. We therefore scale all the other parameters accordingly, i.e., since it is the ratio between the total number of tRNAs and total number of codons which is important, we match this to a real cell. We typically use 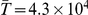. Accurate measurements for the numbers of each individual tRNA species in a real cell are not available for all 41 species; in [31] it is shown that the gene copy number for each tRNA species correlates well with the tRNA abundances where these have been measured. We therefore determine the proportions of each type of tRNA using the gene copy numbers from the *S. cerevisiae* genome (as given in [31]), i.e., 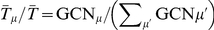, where 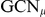 is the gene copy number for the tRNAs of type 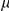. We fix the constant 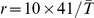 in Eq. (1), such that the mean hopping rate is 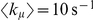, matching that observed experimentally [5].

For the recharging we need values for the constant 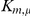, and the maximum charging rate 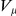, for each synthetase. The maximum charging rate is given by
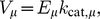
(7)where 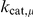, known as the turnover number, is the rate at which one enzyme molecule can recharge one tRNA, and 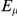 is the number of enzyme molecules present. Measured values for 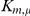 and 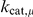 can be found in the literature for some synthetases [32]–[36], but not for all; for this reason, and also because many of the known values are of the same order of magnitude, we take an average of the values from the references above and assume that all enzymes have the same properties. Thus we use a turnover rate of 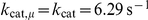, and a value 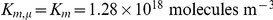. The values used in the calculation can be found in the supporting information (Text S2). 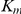 has units of concentration, but since our system has no spatial extent we must convert this into a number of molecules by multiplying via the effective volume - this is the volume a cell would have if it were reduced in size by the same proportion by which we have reduced the number of codons in our system, compared to a real cell. We take 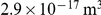 as the actual volume of a typical yeast cell. The number of molecules of each type of enzyme in a typical cell has been measured by Haar [37], and from this data we can calculate the number of enzymes per tRNA molecule; to be consistent with our assumption that all synthetases have the same properties, we use the mean value of 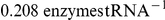.

We use ribosomes of width 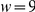 [38]; for convenience it is assumed that it is the rightmost covered codon for which the ribosome is awaiting a tRNA (and this choice does not affect the results [21]).

## Results

### Single Species of mRNA

In this section we consider separately several different mRNAs, and examine the steady state ribosome current and density at different values of the initiation rate 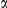. In each simulation we include 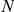 mRNAs, with 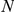 chosen such that there are approximately 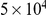 codons in total in the system; the other parameters are scaled as detailed in the methods section. In all cases we find that for small 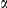 the system is in an LD phase, but as 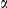 increases above some critical value, queues form behind some codons. In order to understand which codon types are causing these queues we introduce the following quantities: the intrinsic relative speed of the codons
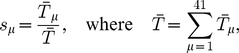
(8)which represents the *supply* of each tRNA type; and representing the *demand* for tRNAs, the relative abundance of the codons
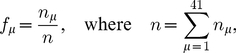
(9)and 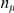 is the number of 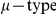 codons on each mRNA.

**Figure 2:**
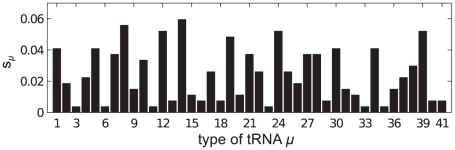
Supply 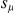
 **for each type of tRNA used in the simulations.** These are based on the gene copy number for each tRNA in the *Saccharomyces cerevisiae* genome. The key for the label 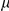 of each codon is available in the supporting information (Text S2).

In the following subsections we examine each mRNA in turn. We label the mRNAs A–D, and list them in table 1; we consider two ribosomal and two other mRNAs. The particular open reading frames which we present have been chosen somewhat arbitrarily, but they are of typical length and codon make up. In each case we match the supply of tRNAs to that of a real cell; Fig. 2 shows the supply 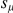 of each tRNA type. The full codon sequence and further information about each mRNA is given in the supporting information (Text S2).

**Table 1:** mRNA sequences used in simulations.

Label	Protein Name	Length	Protein Description
A	YDR382W	110	Ribosomal protein.
B	YLR378C	480	Protein involved in protein secretion.
C	YJL136C	87	Ribosomal protein.
D	YMR307W	560	Protein involved in cell wall biosynthesis.

The mRNA lengths are given in numbers of codons. The codon sequence and further information is given in the supporting information (Text S2).

### mRNA A

**Figure 3:**
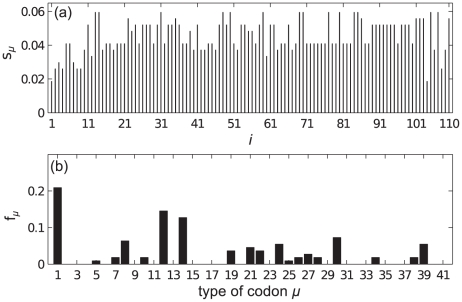
mRNA A Supply and Demand. Bar graphs showing (a) the supply 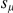 of the codon at each site and (b) the occurrence frequency 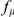 of each codon type on mRNA A.

**Figure 4:**
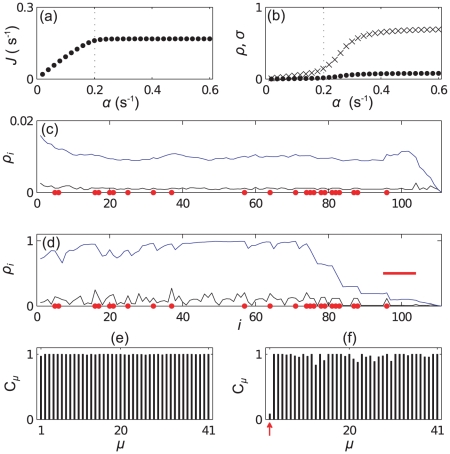
Simulation results for mRNA A. Plots (a) and (b) show how the current (protein production rate) and the mean density of ribosomes on the mRNA depend on the initiation rate 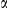, respectively. In (b) the points show the reader density 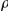 and the crosses the coverage density 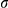. Plots (c) and (d) show ribosome density as a function of position 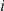 for small (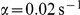) and large (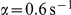) initiation rate respectively. Black lines show the reader density 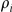 and blue lines the coverage density 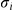. Red dots show the positions of codons of type 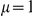, and the red bar indicates the width of the ribosomes. Bar graphs (e) and (f) show the steady state charging rate 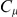 of each tRNA type. (e) shows 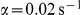 and (f) 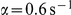, the same values as in (c) and (d).

Fig. 3(a) shows the supply 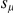 of the tRNA for the codon at each position on the mRNA; this also gives a measure of the intrinsic speed associated with a codon, i.e., it is proportional to the translation rate in the absence of steric interactions and when resources are not limited. The figure can therefore be interpreted as the intrinsic codon speed profile for this mRNA. Fig. 3(b) shows the frequency of usage 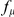 for each codon type, assuming that the whole population of 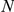 mRNAs are of type A. We note that there are no particularly slow (small 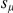) codons, and there is a high abundance of codons of type 1. Figs. 4(a) and (b) show how the current and density vary with 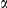; we see that initially 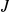 and 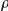 (and also 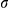) increase with 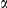, before reaching a plateau - a profile strikingly similar to that of a simple mono-codon mRNA [20]. In Figs. 4(c) and (d) we plot the density profile, i.e., the time average occupation of each site 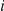, for two different values of 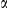 respectively. We again consider two different measures of density: the reader density 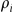, i.e., for the rightmost site covered by a ribosome only, and the total coverage density 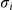. As one might expect, the coverage density is approximately 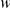 times the reader density; however we shall see below that different features can sometimes be seen in each kind of profile. From these figures we see that for small 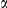 the system is in the LD phase, but for an initiation rate above some critical value 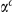 we have behaviour which approximates a queue to the left of codon 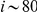. We highlight the different scales on the vertical axis of the two plots. Queueing is consistent with the experimental observation [8] that on average the density of ribosomes decreases along the mRNA.

The queue might seem surprising given the information in Fig. 3(a) alone, as there are no especially slow codons near 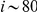. In Figs. 4 (e) and (f) we plot the steady state relative charging level of each tRNA type, which we define as
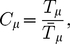
(10)where here 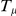 is the steady state average number of charged tRNAs and, as before, 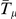 is total number of 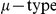 tRNAs (charged and uncharged). The plots shown are for the same two values of 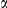 as in 3 (c) and (d). We note that the charging level of tRNAs of type 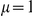 has decreased significantly at large 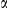, i.e., due to the finite recharging rate and high demand for that tRNA type, 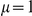 codons have become slow codons. Examining the mRNA sequence shows several clusters of type 1 codons around site 80, which are responsible for the queue (marked as red dots in Figs. 4(c) and (d)). This is consistent with previous work [39] which shows that the effect of slow codons is greatly enhanced when they appear in clusters.

**Figure 5:**
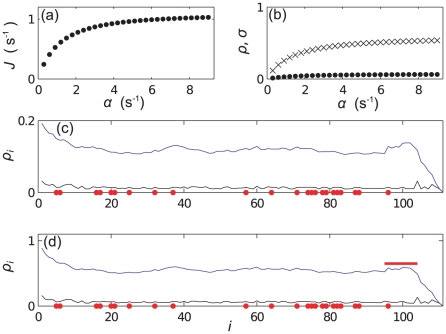
Simulation results for mRNA A using the original TASEP model. The tRNA charging rate is assumed to be infinite, and hence then numbers of aa-tRNAs are constant and based on gene copy numbers. In (b) the points show the reader density 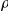 and the crosses the coverage density 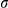. Plots (c) and (d) show ribosome density as a function of position 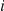 for small (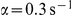) and large (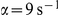) initiation rate respectively. Black lines show the reader density 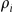 and blue lines the coverage density 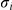. Red dots show codons of type 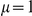 as in Fig. 4.

In summary, the aa-tRNA species for which there is most demand (largest 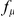) becomes depleted for large 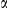, leading to queueing behind clusters of this type of codon. In Fig. 5 we show similar results for a model where all tRNAs are assumed to be charged at all times (i.e., the 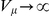 limit) and the 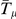 are based on gene copy numbers (i.e., tRNA supply) only, as has been assumed in previous work. We note that the behaviour is very different; as there are no particularly slow codons, the system does not display queueing; instead it reaches a maximal current (MC) due only to the steric repulsion between the ribosomes. We also find that even if the 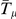 are rescaled so as to take into account the demand as well as supply of tRNAs, the results are still different from those of the more complete model presented here (results not shown).

### mRNA B

**Figure 6:**
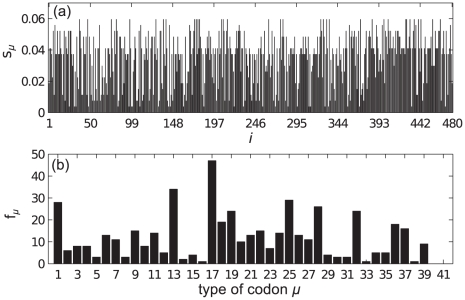
mRNA B Supply and Demand. Bar graph showing (a) the supply 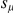 of the codon at each site and (b) the occurrence frequency 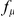 of each codon type on mRNA B.

**Figure 7:**
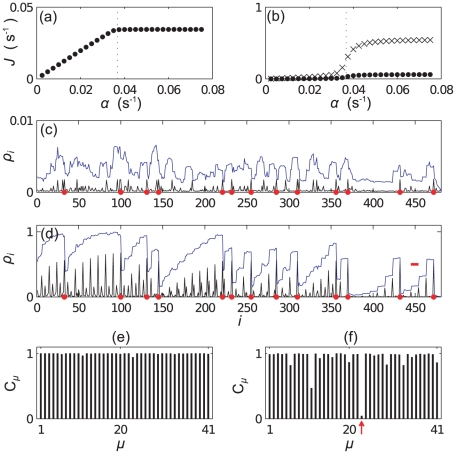
Simulation results for mRNA B. Subplots are as described in the caption for Fig. 4. Plots (c) and (d) show density for small (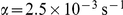) and large (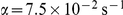) initiation rate respectively. Red dots show the positions of codons of type 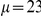, and the red bar indicates the width of the ribosomes. (e) and (f) show 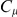 for 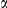 the same as in (c) and (d).

Fig. 6 shows plots of 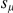 for each site on a type B mRNA, and the abundance 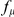 of the different codon types now assuming that all of the 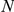 mRNAs are of type B. In contrast to mRNA A, here there are many low 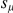 codons distributed throughout the mRNA. Fig. 7 shows results analogous to those for mRNA A. We see from Fig. 7(f) that here it is the charging level of tRNAs of type 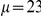 which becomes most depleted at large 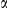, and observe queueing behind codons of this type. In the density profile at large 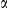 (Fig. 7(d)) we note that not only are queues clearly visible, but also that there is some periodic structure in the profile for both of the density measures. The peaks in the reader density and the features in the coverage density are caused by the extended volume of the ribosomes, and have a width equal to that of the ribosomes - 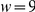 codons (this length is indicated by a red bar in the figures). This was not observed for mRNA A because the slow codons appeared in clusters and were separated by distances less than 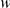 - the effect was smeared out. In mRNA B the slow codons (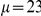, shown as red dots) are separated by much larger distances, and queues are found behind each. Another interesting feature of the density profile in Fig. 7(d) is the shape of the profile immediately to the left of the slowest codons: queues towards the right side of the mRNA usually show a concave decay (e.g. left of codon 432), whilst some queues towards the left side show a convex decay (e.g. left of codon 221). Previous work on sequences which only contain two different codon species [40] suggest that these features depend somewhat on the width of the regions between the slowest codons, as well as the elongation rates of these codons, but this is far from fully understood and is beyond the scope of the current work.

**Figure 8:**
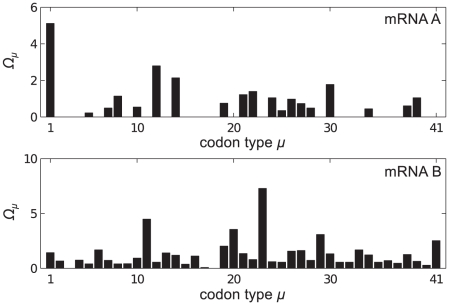
The demand supply ratio. Bar graphs showing the ratio 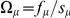 for each codon type for mRNAs A and B.

The situation for mRNA B further differs from that of mRNA A because codons of type 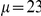 (the slow codons) do not have a high 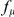 value. To explain this behaviour we introduce the quantity
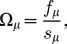
(11)which is the ratio between the demand for and supply of tRNAs, and is a measure of a type of codon's propensity to cause queueing. Fig. 8(b) shows this for mRNA B, and we note that 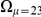 has the largest value. From comparison with Fig. 7(f) we find that 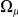 is also an indicator of how the charging level of tRNAs of type 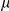 will be affected. Fig. 8(a) shows 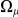 for mRNA A, correctly identifying codons of type 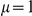 as those which become rate limiting.

### Estimation of 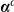


It is clear that the queueing behaviour arises because one of the aa-tRNA species has become depleted, i.e., we have entered a 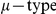 limited resources (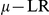) regime. This is characterised by a reduction in 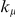 (as shown in Figs. 4(e) and (f) and 7(e) and (f)) at some critical initiation rate 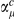 where the rate at which 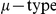 tRNAs are being used (which we denote 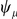) reaches the rate at which they can be recharged (denoted 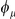). The critical initiation rate for queueing is therefore
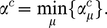
(12)


Consider the LD regime where we assume that the current depends on the average supply of each tRNA type (in this regime tRNAs can be assumed to be fully charged), i.e.,
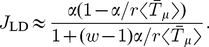
(13)The angled brackets denote the average over 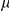. The rate at which 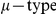 aa-tRNAs are used is therefore
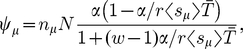
(14)where as before 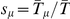 (Eq. (8)), 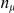 is the number of 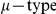 codons on each mRNA, and 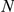 is the total number of mRNAs. Notice that by definition 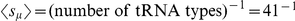. From Eq. (3) the maximum recharging rate for 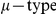 tRNAs is
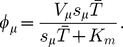
(15)Equating 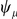 and 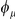 gives the critical initiation rate for tRNAs of type 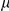


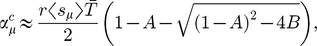
(16)where
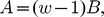


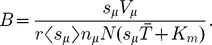
The 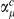 with the smallest value gives a reasonable estimate for 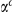, shown as dotted vertical lines in Figs. 4(a) and (b), and 7(a) and (b). From Eq. (16), by assuming 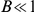 (which is true for realistic parameters) and expanding to first order, we also find 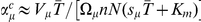, i.e.,
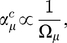
(17)which is consistent with the observation stated in the previous subsection that the codon species associated with the largest value of 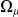 (as defined in Eq. (11)) causes queueing.

In both of the examples above we see 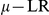 induced queueing. In the first case (mRNA A) an otherwise averagely abundant aa-tRNA becomes depleted due to the high usage frequency 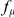 of that codon type. In the second case it is an intrinsically slow codon which leads to queueing. These two different types of behaviour show that even in a simulation with only one type of mRNA, the dynamics are highly sensitive to the precise usage of codons.

### mRNAs C and D

We have investigated two further examples of mRNAs treated individually with tRNA supply matched to that of a real yeast cell as before. The results are very similar to those of mRNAs A and B, so we include these as supporting information (Text S3). One slight difference is that the point of the onset of the 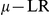 induced queueing is less well defined than in the previous cases. It has already been documented that slow codons appearing in close proximity to the initiation site can lead to a smoothed onset of queueing [15], but here there is an additional effect in that there are several codon species which become depleted. That is to say more than one codon species acts as a bottleneck, and the 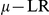 regime is entered at slightly different values of 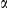 for each.

## What is the Nature of the Queueing?

**Figure 9:**
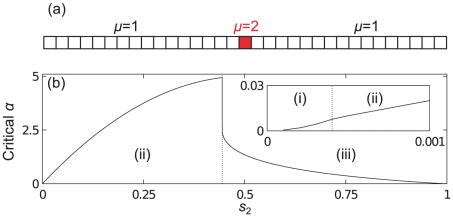
Different reasons for queueing in a “designer mRNA”. (a) Sketch of a designer mRNA with only two types of codon. All codons are the same except for the central one. (b) The solid curve shows the critical initiation rate beyond which queueing will be observed, as a function of 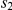. Which kind of queueing will be observed depends on 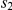, and the three regimes discussed in the text are separated with dotted lines. The inset shows a zoom around small 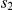.

In this subsection we use a very simple “designer mRNA” to help explain the nature of the queueing regime. We consider a system with only two types of codon and tRNA, with an mRNA sequence of length 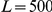 where all codons are of type 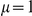, except the central codon which is of type 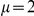; i.e., 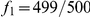, and 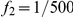 (where 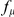 is defined in Eq.(9)) This is shown schematically in Fig. 9(a). We consider the different regimes as the initiation rate 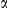 is increased whilst, as before, assuming that the termination rate is not limiting (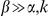).

In the original TASEP (the 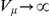 limit), where hopping rates have fixed values 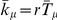, there are two possibilities as 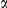 is increased: if 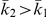 there is a smooth transition from an LD to a maximal current (MC) phase; if in contrast 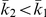 there is a sharp transition from LD to a queueing phase (QP), where ribosomes queue behind the 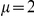 site [11], [23]–[27]. The system is in a QP for initiation rates larger than
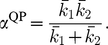
(18)


In the finite recharging model we have observed a third possibility. One or more of the tRNA types can start being used up at a rate comparable to the recharging rate, i.e., its charging level is reduced and it becomes a limited resource: there is 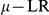 induced queueing. As detailed above, we can calculate an approximation for the initiation rate 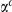 at which the system will move into this regime. Queues of ribosomes build up behind 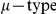 codons; crucially, since the onset of 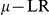 is smooth, the onset of queueing will also be smooth. There is therefore a clear difference between 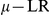 induced queueing and a QP transition. It has been shown in [20] that for realistic recharging parameters, the LR regime is always reached before the MC phase.

For our toy mRNA sequence, whether we observe 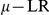 or QP depends on which transition is reached first. In this case Eq. (18) gives 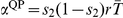. Fig. 9(b) shows 
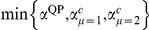
 as a function of 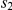 (where 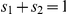 since we have only two codon types). We obtain three regions, as labelled in the figure:

In this narrow region – shown in the blow-up on the right of the figure – 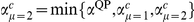; hence the system will reach a 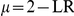 induced queueing regime when 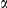 is above the shown critical value. Therefore there is a queue behind the 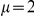 codon. As 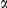 is increased through the critical value the smooth 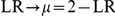 induced queueing transition occurs.

In this region 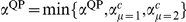, and hence the system reaches a QP for initiation rates above the critical value shown. Ribosomes will queue behind the 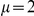 codon (due to the low value of 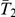) without any tRNAs becoming limited. As 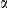 is increased through the critical value there is a sharp 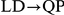 transition. At higher values of 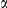 there may be a further LR regime within the QP, but the point at which this regime is entered cannot be estimated in the framework discussed here; instead we refer the reader to Ref. [20].

In this third region 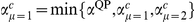, and therefore it is the 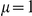 codons which become depleted first, and the system enters a 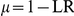 regime for initiation rates above the shown critical value. There is a 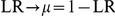 transition as 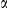 is increased. Notice that there is no queueing since the slow codons make up the bulk of the mRNA, including at the beginning of the sequence [15].

For a real mRNA sequence, since there are many tRNA types, the 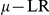 regime will most likely lead to queueing. Although in theory it is possible to reach a real QP transition before any tRNAs become limited, this has not been observed for any realistic mRNA sequence analysed.

## Mixtures of Multiple mRNA Species

In this subsection we consider simulations which contain multiple types of mRNA attached to the same pool of tRNA resources. As before we choose the tRNA abundances such as to match the *supply* of a real cell. The *demand* on those resources depends on the proportion of each type of codon in each type of mRNA, and the proportions of each type of mRNA. We first present two example systems, each containing multiple copies of one short mRNA and multiple copies of one long mRNA. Finally we present simulation results from a system containing 70 different mRNA species.

### Mixtures of mRNA A and mRNA B

Here we show the effect of varying the initiation rate on the ribosome current, density, and tRNA charging level, for several different mixtures of mRNAs of types A and B (with lengths 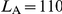 and 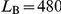 respectively). We use the same initiation rate for each mRNA. We examine systems with (i) an equal amount of each mRNA *by codon* (i.e., there are the same number of codons in all of the mRNAs of type A as there are in all of the mRNAs of type B), (ii) with the number of codons in type A mRNAs having the ratio 20% to 80% of those in type B mRNAs, and (iii) the ratio 80% to 20% type A to type B by number of codons. In each case we include approximately 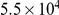 codons in total (scaling the parameters accordingly as in earlier sections). This means that in each case we have

- 50∶50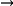 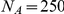 and 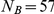,
- 20∶80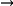 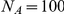 and 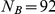,
- 80∶20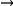 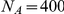 and 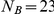,

where 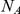 and 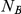 are the numbers of mRNAs of type A and B in the system respectively. Comparing these with the numbers of mRNA copies found in a real cell (see supporting information Text S2), the 80∶20 proportion is the most realistic.

**Figure 10:**
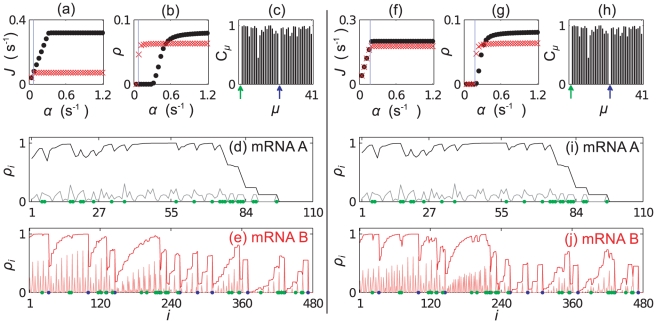
Results for simulations containing mixtures of mRNAs A and B. Plots (a)–(e) show results for a mixture in the ratio 50∶50 (by codon numbers). (a) and (b) show 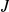 and 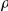 as a function of 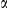 for mRNAs of type A (black points) and type B (red crosses) (the same initiation rates are used for each species). The blue line shows 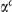, where blue labelled codons cause queueing. Plot (c) shows the charging levels of tRNAs for 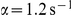. (d) and (e) show the site dependent reader (pale lines) and coverage (dark lines) density for each mRNA type, again for 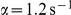. The codons corresponding to the first aa-tRNA to become depleted are highlighted with blue dots (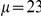), and those for the second in green (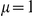). Plots (f)–(j) show similar results for a mixture in the ratio 80∶20; results for a 20∶80 mixture are presented in the supporting information (Text S4).

Figs. 10(a)–(e) and 10(f)–(j) show simulation results for the 50∶50 and 80∶20 mRNA mixtures respectively. In each case we plot the current 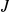 (which corresponds to the protein production rate *per mRNA*) and the reader density 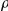 as functions of 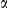, and the charging levels of each tRNA 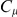 (defined in Eq. (10)) for the largest value of 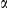 investigated. We also show the reader and coverage density as a function of position for large 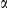 (during 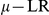 induced queueing) for each mRNA. In each case we indicate the 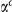 where the first tRNA species becomes depleted, and indicate the positions of these codons (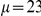) with a blue dot. The critical initiation rate can be estimated as before, but now the 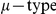 tRNA use rate is given by
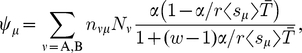
where we sum over mRNA species, and 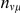 is the number of 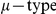 codons on type 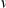 mRNAs. This equation assumes that the LD current is the same through both types of mRNA, i.e., it assumes the average 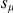 of the codons on each mRNA is approximately equal to 
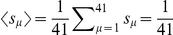
.

**Figure 11:**
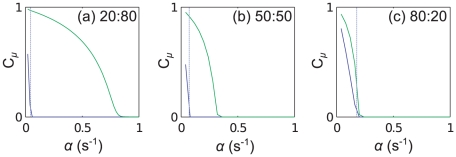
The charging levels 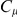
 **of the first two aa-tRNAs to become depleted.** Plot (a) shows results for the 20∶80 mixture of mRNAs A and B, plot (b) the 50∶50 mixture, and (c) the 80∶20 mixture. From left to right the abundance of mRNA A increases. Blue and green lines correspond to the codons labelled blue and green in Fig. 10, and dashed lines show 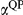.

We note that only the long mRNA B contains the “blue” codons; in each case the current on mRNA B reaches a maximum at 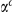 due to “blue”-LR induced queueing (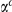 is indicated by a blue vertical line in the figures). Since mRNA A does not contain these queueing codons, the current there (denoted 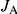) continues to rise; the sharp change in 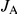 at a larger 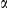 indicates a queueing transition rather than a maximal current transition [15]. We do indeed see that a second tRNA species (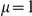) also becomes depleted (indicated in green). In the case of the 50∶50 ratio of A to B the transition to queueing in mRNA A is at an initiation rate several times 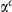, whereas in the 80∶20 case, the transition is at just slightly greater than 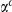. The 20∶80 mixture shows results qualitatively the same as the 50∶50 mixture, so for conciseness we present those results in the supporting information (Text S4). In Fig. 11 we show the charging level of the marked tRNA types as a function of 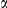; in each case we see the relatively sharp reduction of the charging level of the blue tRNAs at 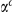. For the 50∶50 and 20∶80 cases there is a much more gradual decrease in the charging level of the green tRNA, whereas in the 80∶20 case (where there is an abundance of mRNA A which contains the green labelled codon) the decreases is much sharper. We cannot use the above method to estimate where the second queueing transition will occur, since as soon as queueing starts on one mRNA species, the current can no longer be estimated using Eq. (13).

### Mixtures of mRNA C and D

We now look at simulations with different mixtures of mRNAs C and D; again one is short (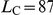), and the other is much longer (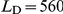), but here we find some quite different results to those discussed above. We consider three different simulations with the following proportions by number of codons of each mRNA type

- 50∶50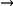 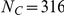 and 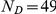,
- 20∶80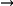 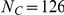 and 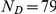,
- 80∶20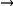 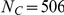 and 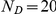,

where here the 20∶80 mixture is the closest to a real cell when considering the mRNA copy number (see supporting information Text S2).

**Figure 12:**
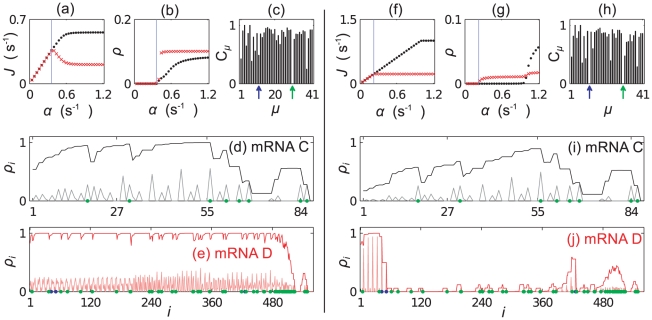
Results for simulations containing mixtures of mRNAs C and D. Plots (a)–(e) show results for a mixture in the ratio 50∶50 (by codon numbers). (a) and (b) show 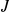 and 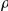 as a function of 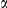 for mRNAs of type C (black points) and type D (red crosses). The blue line shows 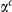, where blue labelled codons first become depleted. Plot (c) shows the charging levels of tRNAs for 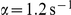. (d) and (e) show the site dependent reader (pale lines) and coverage (dark lines) density for each mRNA type. The codons corresponding to the first aa-tRNA to become depleted are highlighted with blue dots (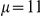), and those for the second in green (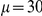). Plots (f)–(j) show similar results for a mixture in the ratio 20∶80; results for a 80∶20 mixture are presented in the supporting information (Text S4).

**Figure 13:**
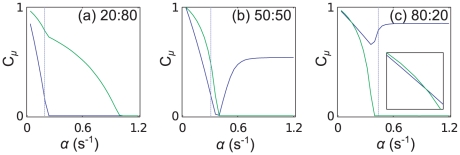
The charging levels 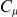
 **of the first two aa-tRNAs to become depleted.** Plot (a) shows results for the 20∶50 mixture of mRNAs C and D, plot (b) the 50∶50 mixture, and (c) the 80∶20 mixture. From left to right the abundance of mRNA C increases. In each case the tRNA type labelled blue becomes depleted first. The inset in (c) is a zoom at small 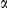 showing this more clearly. Dashed lines show 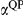.

In Figs. 12(a)–(e) we present results for the case where there is an equal number of codons in all mRNAs of type C and in all mRNAs of type D; the aa-tRNA type which becomes depleted first (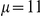, labelled blue) is only present on the long mRNA. As in the previous section, even once queueing begins to occur on that mRNA, the current of ribosomes along the short mRNAs continues to increase. A strikingly different outcome here is that the current through the long mRNA then begins to decrease again. This happens because initially a queue forms behind the blue codons near the beginning of mRNA D; since there are no blue codons on mRNA C, the current there continues to increase with 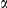. As 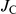 increases, a second type of tRNA (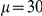 labelled green) becomes depleted. The large cluster of green codons near the end of mRNA D begins to cause a more serious queue than the two blue codons near the start - the current along mRNA D decreases. This decrease leads to a lower rate of blue tRNA use, and the charging level therefore increases. This can be seen in Fig. 13(b).

Consider now decreasing the numbers of mRNA C compared to D, i.e., consider the 20∶80 C to D mixture (Figs. 12(f)–(j)). There are now more blue codons, but the same number of blue tRNAs. The blue codons become queueing at first, and as before 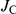 continues to increase. Although the green codons are again the second species to become depleted, this time the codons are not slower than the blue codons. The demand for green codons is not sufficient to make the queue behind the large green cluster on mRNA D more severe than the queue behind the blue codons. At the second transition a slight increase in the density on mRNAs of type D is seen - Fig. 12(g); this is because although the green codons are not the slowest codons, there is a slightly increased density behind them (e.g., at several points between 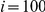 and 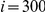 in Fig. 12(j)).

If we increase the number of type C mRNAs (the 80∶20 C to D mixture), we have a different outcome again. The situation is very similar to that of the 50∶50 mixture, but now the demand for green tRNAs (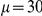) is so large, that these become depleted almost immediately after the blue tRNAs as 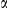 is increased. These results are presented in the supporting information Text S4. The reduction of the current through mRNA D is so severe that the charging level of blue tRNAs returns almost to full. This can be seen in Fig. 13(c).

We have shown that changing the relative numbers of mRNAs can be very important in determining the dynamics of the system. We match the tRNA supply to that of the real cell, and even though the considered demand is not realistic, we show that different patterns of codon usage can lead to very different behaviour in terms of protein production rate. Therefore we have demonstrated that protein production can be controlled at the translation elongation level by means of the interplay between demand and supply of tRNAs. This is likely to be highly important since levels of different mRNAs are likely to be vary for many reasons, e.g., as a response to environmental stress, or throughout the different phases of the cell cycle.

#### *Larger scale simulations*

We now present results from some larger scale simulations which contain many mRNA species. Due to computational limitations a detailed analysis of such systems is not possible, however we are able to show that the balance between supply and demand is still important even in much larger systems.

We take as an example the fact that during different phases of the cell cycle around 15% of genes display significant changes in expression [41]. We choose a selection of 10 mRNAs which are known to change from a high to a low abundance (or vice versa) between the G1 phase and the G2 phase of the cell cycle; we refer to these as group I mRNAs and list them in table 2. We then include these in a simulation alongside 60 other mRNA species (the levels of which are thought to remain constant), such that the group I mRNAs make up 15% of the total. The 60 other mRNAs are chosen arbitrarily, with 20 highly abundant, 20 medium-highly abundant and 20 medium abundance mRNAs (abundance data from [29]); we refer to these as group II mRNAs. As before we consider a reduced system, this time containing a total of around 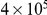 codons; the number of each of the group II mRNAs are chosen such that they have the same relative abundance as in a real cell. We perform two simulations, one where the group I mRNAs have either high or low abundance as they would in G1, and the other with abundances as in G2 (see table 2). The number of each group II mRNA is kept the same in each case. Full details of the mRNAs are given in supporting information Text S2. As in previous sections we choose tRNA abundances such that the proportions of each species match those of a real cell (based on tRNA gene copy numbers [31]). The total number of tRNAs is chosen so that the ratio of this to the total number of codons is the same as is found in a real cell. For this calculation we assume the total number of codons is the average of the two simulations, and use the same numbers of tRNAs for each. The abundance of each group II mRNAs is chosen so that the proportion of each matches that found in a typical cell [29]. Since the typical abundance of the group I mRNAs found during each cell cycle phase is not well known, we match the abundances to the most and least abundant group II mRNAs. Due to computational limitations we cannot run simulations for a range of initiation rates, so we make a crude estimate of 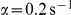 (based on a translation rate of 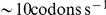 [5] and an inter ribosome reader separation of 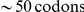 [17]), and assume this is the same for all mRNAs.

**Table 2:** Group I mRNA sequences used in large scale simulation.

Label	Protein Name	Length	Level in G1	Level in G2
1	Cln3	581	High	Low
2	Cdh1	567	High	Low
3	Cdc20	611	Low	High
4	Clb1	472	Low	High
5	Clb6	381	High	Low
6	Sic1	285	High	Low
7	Cln1	547	High	Low
8	Cln2	546	High	Low
9	Clb2	492	Low	High
10	Clb5	436	High	Low

A selection of 10 mRNA which are known to change their expression level between the G1 and G2 phases of the cell cycle. Lengths are given in numbers of codons.

**Figure 14:**
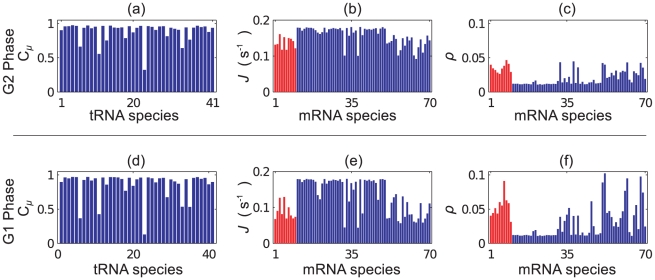
Simulations of large numbers of mRNAs. Results from two simulations containing 70 different species of mRNA (numbered 1 to 70 with full details being given in supplementary information S2). In both simulations the abundance of each of group II mRNAs (blue) are kept the same, but the abundance of group I mRNAs (red) are varied so as to match their abundance during the G1 and G2 phases of the cell cycle respectively. Plot (a) shows the charging levels for each tRNA, (b) the current for each mRNA and (c) the corresponding ribosome densities, each for G2 phase. Plots (d)–(f) show similar for G1 phase.

In Fig. 14 we show results from the two simulations; we show bar graphs of tRNA charging levels and the currents and average ribosome density for each mRNA species. Note that the current is equivalent to the protein production rate per mRNA. We observe that there is a significant change in the charging level of some tRNA species. This leads to large changes in the current of some mRNA species, whilst others a largely unaffected. The protein production rate per mRNA changes by more than 50% in some cases. Similar patterns of change are also seen in the ribosome density. We note that in general those mRNA which contain copies of the codons for which the tRNAs become most depleted show the largest change in density. Interestingly the total ribosome usage for the G1 simulation is 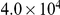, which is more than double the value of 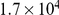 in the G2 simulation; these values equate to 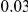 and 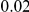 ribosomes per codon, respectively.

In these simulations we do not include a completely realistic demand for tRNAs, but in taking a representative subset of mRNAs we show that the balance between demand and supply plays an essential role in translational regulation of gene expression. These results do show that relatively small changes in demand (of the scale a real cell will experience during its normal life cycle) can have a large effect on the production of some proteins.

## Discussion

In this paper we have shown that it is the interplay between demand and supply which determines the existence of bottlenecks in translation elongation, and that this could be used by the cell to control protein production. We apply a recent model of the elongation step of mRNA translation, which includes the dynamics of the use and recharging of aa-tRNAs, to realistic mRNA sequences from the *Saccharomyces cerevisiae* genome. We show that that including the use and recharging of aa-tRNAs in the model has a significant effect on the dynamics. We obtain a regime where a particular tRNA type becomes depleted leading to the relevant codons becoming “slow”, and causing queueing. Whilst previous authors [15], [27] have assumed that it is the type of codon associated with the tRNA with the lowest abundance which is most important for queueing, we have shown that which one of the codon types is (or becomes) the slowest depends both on the supply of (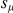), and the demand for (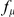) the relevant tRNA species (defined in Eqs. (8) and (9)). We have also found that merely taking both supply and demand of tRNAs into account in a TASEP model does not give the same results as fully describing the dynamics of the recharging process as we have done here.

In the simulations we choose the supply of tRNAs based on the numbers of each tRNA type found in a real cell, i.e., we have matched the supply of tRNAs to that of a real cell. At low initiation rate, when none of the tRNA charging levels are significantly reduced, the slowness of each codon type depends only on the tRNA supply. At high initiation rate, for some tRNA types the charging levels become reduced: which ones depends on the demand for each type of tRNA.

In the case of simulations where only one type of mRNA is included we have shown that the behaviour can be predicted by considering the quantity 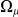, defined as the ratio between the tRNA demand and the supply (Eq. (11)). The value of 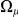 can be used to predict which codons will be the first to become queue causing as the initiation rate is increased. This might lead one to ask whether a full dynamic treatment of recharging is really necessary. We investigated this hypothesis using a model with fixed hopping rates (the original TASEP), choosing 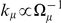 (data not shown). Although we saw queueing behind the same type of codon as in the results presented here, this was obviously due to a QP transition rather than 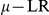 induced queueing. Also the onset of the regime was at a different initiation rate - e.g. for mRNA B in the model with fixed hopping rates this was on the order 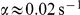, compared to 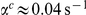 in the results presented here. Whilst this is only a minor difference in the case of single mRNAs, none of the interesting “competition” effects observed in the case of simulations with multiple mRNA types would be observed in a model with fixed hopping rates.

Since in each simulation we treat a small subset of mRNAs, the demand for tRNAs is not the same as in a real cell. We conclude that in situations where the demand is important, it is difficult to predict the effect on protein production from a specific mRNA without considering the entire mRNA set, which is a computationally ambitious task. Nevertheless, we have shown that the interplay between demand and supply is what determines which codons become rate limiting for translation. Other authors [13] have attempted to treat real mRNA sequences using an iterative mean field approach. If this could be combined with our model of finite recharging, it could offer significant improvement to brute force simulation of the entire genome.

One might consider comparing values of 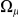 with known measures of slow codon usage such as the codon adaptation index (CAI) [42]. The CAI is a property of an mRNA sequence and is a measure of the translation efficiency, or more precisely the synonymous codon usage bias of the sequence. It is calculated based on a quantity known as the relative synonymous codon usage (RSCU). The RSCU for codon species 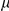 which encodes for amino acid species 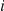 is defined 
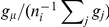
, where 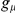 is the total number of codons of species 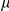 within a set of highly expressed genes [42], 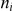 is the number of species which encode for amino acid 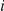, and the sum is over all of these species. The maximum RSCU value for a given amino acid is denoted 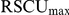. The CAI for a given mRNA is given by the geometric mean of 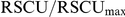 for each codon in the sequence. Our quantity 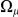 is a similar measure to the RSCU in that it also measures the translation efficiency of a codon, but with respect to how likely that codon is to cause queueing. We can therefore compute a new index for an mRNA by taking the geometric mean of the 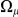 for each codon in an mRNA, i.e. 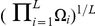 where the product is over all 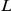 codons in the sequence. We term this the queueing likelihood index (QLI). A comparison between this and the CAI is given in the supporting information Text S5. We find that there is a strong correlation between these quantities (a Pearson correlation coefficient of −0.808), so the QLI can also be used as an alternative measure of translation efficiency. The strong correlation is expected since both quantities use codon usage data; the QLI differs from the CAI in that is explicitly includes tRNA availability data as well as codon usage. An additional advantage of the QLI is that it gives a prediction of how translation will be effected by *changes* in supply or demand.

In a real cell the demand for tRNAs changes throughout the cell cycle, both due to different patterns of transcription, and via mechanisms such as storage, release and degradation of mRNAs in P-bodies [43]. Although small changes in the levels of, for example, a single mRNA are unlikely to have a major impact on the total tRNA demand, we would expect that significant changes in demand would result from, for example the 15% of mRNAs which change their expression level between the G1 and G2 phases of the cell cycle [41]. We have presented simulation results that, although still only treating a small subset of mRNAs, show that a change in mRNA abundances of this magnitude can significantly alter the production rate of some proteins.

We can also apply our analytic treatment to estimate the initiation rate at which the first tRNA species will become depleted. By assuming that all mRNAs have the same initiation rate, and using measured data for the mRNA abundances in a typical cell [29] along with tRNA gene copy number data [31], we can calculate 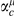 for each tRNA species using Eq. (17). We find that some tRNAs will never become depleted (i.e., another codon type will become rate limiting first), whilst those most likely to become bottlenecks include *Leu5* with 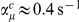 and *Gln2* with 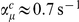. A crude estimate of a typical initiation rate of 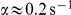 (based on a translation rate of 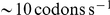 and an inter ribosome reader separation of 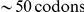 [17]) allows one to speculate that a two-fold increase in the initiation rate may be enough to cause queueing. Such an increase in the initiation rate could be achieved through for example a nutrient up-shift leading to ribosome biogenesis up-regulation.

We have shown in this paper that changes in the supply and demand can drastically alter the behaviour of the protein production mechanism, and different patterns of slow codon usage can act as a means for control. It is known that control of protein production rates is also exercised heavily at the initiation stage of translation, via, for example, structure in the 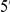 untranslated region which varies across different mRNAs [2], or more globally through regulation of initiation factors such as eIF2 [44]. It is also thought that variation of initiation rates across different mRNAs is used to effect “translation on demand” [1]. We show here how changes in the initiation rate could be used in conjunction with changes in supply and demand of tRNAs, for example to move from queueing to non-queueing behaviour. Other feedback mechanisms which could be executed by the cell to prevent charged tRNA depletion include production of extra aminoacylation enzymes. Another consideration is that in a real cell the availability of ribosomes could be an important factor: a typical cell contains of the order 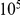 ribosomes [37], which is about 0.05 per ORF codon; in our simulations for queues the mRNA coverage can reach around 0.08 ribosomes per ORF codon in the case of simulations with one or two mRNA species, or 0.03 ribosomes per ORF codon in the larger scale simulations. If significant numbers of mRNAs in a cell were to have queues, the amount of free ribosomes in the cytoplasm could become depleted likely leading to a reduction in initiation rate - this itself could act as a feedback to reduce queueing. Finite numbers of ribosomes have previously been considered in a TASEP model [7], but not in the biological context of finite tRNA recharging.

In our simulations we ignore the effect of wobble base pairing. It is known that some tRNAs can still recognise a codon when only the first two of the three nucleotides match correctly; the cost of this mismatch is that the hopping rate for such codons reduces by approximately one third [45]. Some authors [46] compensate for this effect in models by rescaling the number of tRNAs for the “wobble” tRNAs; as the current work has shown, the number of tRNAs is crucial to the dynamics, so we do not follow this strategy here. A more realistic approach would be to have a codon type dependent intrinsic hopping rate 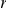. Other improvements which could be made to the current model include considering multiple internal states for ribosomes [47], or using a more realistic model for aminoacylation which considers the differences between each enzyme, and takes into account the availability of each amino acid. A reformulation of Eq. (3) to more realistically describe an enzymatic reaction with multiple substrates (such as in [48], [49]) could the allow amino acid starvation conditions to be studied in this framework. We also do not consider here effects such as so called “no-go decay”, where mRNAs upon which there are stalled ribosomes are selectively degraded [50]. This could be considered a feed back effect to release resources. A phenomena related to no-go decay is ribosome drop off, the probability of which increases due to stalling at slow codons [51]; this could also be incorporated into future models, although since it occurs at a low rate and in yeast is more likely to be due to secondary structure than slow codons [51], it is unlikely to qualitatively change the behaviour.

In summary, when this recent model is applied to realistic mRNA sequences we find queueing behind slow sites or clusters of slow sites. The present model differs from previous ones in that the particular species of codon which becomes slow depends on the demand placed on aa-tRNAs and not just the overall tRNA abundances. We find that the behaviour depends on the dynamics of the system, and the same results cannot be produced with constant hopping rates; i.e., including the full charging process in the model is crucial. We have shown that in larger systems, changes in the demand for tRNAs which occur during the cell's normal life cycle are sufficient to cause significant changes in protein production.

## Supporting Information

Text S1 We present the derivation of our TASEP based model of translation which includes both extended particles and finite tRNAs in a uniform mRNA.
(PDF)

Text S2 Includes the key for the labelling of codon/tRNA species, the values for enzyme kinetic constants used, and the sequences and description of each mRNA sequence given in table 1. Also includes the full list of mRNA species used in the larger scale simulations.
(PDF)

Text S3 Includes results for simulations of mRNA C and D individually.
(PDF)

Text S4 Includes further results for simulations of mixtures of mRNAs A and B, and mixtures of mRNAs C and D.
(PDF)

Text S5 Comparison of the CAI and the QLI.
(PDF)
