# Supplementary material for: The Dynamics of Supply and Demand in mRNA Translation
Source: PLoS Comput Biol. 2011 Oct 13;7(10):e1002203. doi: 10.1371/journal.pcbi.1002203 (PMC3192816; doi:10.1371/journal.pcbi.1002203)
Supplement: Text S1 — We present the derivation of our TASEP based model of translation which includes both extended particles and finite tRNAs in a uniform mRNA. (PDF) [file pcbi.1002203.s001.pdf]

The Dynamics of Supply and Demand in mRNA Translation

Supporting Information Text S1:

Derivation of a TASEP based model of translation of a uniform mRNA which includes both extended ribosomes and a finite charging rate for tRNAs.

Chris A. Brackley[1*], M. Carmen Romano[1,2], Marco Thiel[1]

1 *Institute for Complex Systems and Mathematical Biology, SUPA, University of Aberdeen, Aberdeen, AB24 3UE, UK*
2 *Institute of Medical Sciences, Foresterhill, University of Aberdeen, Aberdeen, AB25 2ZD, UK*

In this paper we consider a TASEP model of translation in which there are a finite number of tRNAs, meaning the elongation rate is dynamic; we also include implicitly the fact that ribosomes obscure several codons as they move along the mRNA. For the case of uniform (mono-codon) mRNAs, models with finite tRNAs and extended ribosomes have been examined in isolation (in [1, 2] and [3, 4] respectively); here we present a derivation for the model which includes both of these features.

In [3] for a TASEP with particles (ribosomes) of length w the authors calculate the current J in the low density (LD) phase by considering the occupation probability of each of the first w sites; similarly in the high density (HD) phase - where exit from the lattice is the limiting process - J is calculated by considering the occupation probability last w sites. For the maximal current (MC) phase they treat the system as a 1D gas of hard rods on a discrete lattice (a discrete Tonks gas) and calculate the current by writing down the partition function for this system. They assume a constant hopping rate k, giving the following equations

$$\text{LD}: \quad J = \tfrac{\alpha(1-\alpha/k)}{1+(w-1)\alpha/k}, \quad \rho = \frac{\alpha/k}{1+(w-1)\alpha/k} \tag{1}$$

$$\text{HD}: \quad J = \tfrac{\beta(1-\beta/k)}{1+(w-1)\beta/k}, \quad \rho = \frac{(1-\beta/k)}{1+(w-1)\beta/k} \tag{2}$$

$$\text{MC}: \quad J = \tfrac{k}{(1+\sqrt{w})^2}, \quad \rho = \frac{1}{w+\sqrt{w}}. \tag{3}$$

Since these have been derived using probabilistic arguments, to include finite number of tRNAs we can directly substitute the constant k by the charged tRNA dependent rate $k \to k(T(t))$. From [1] the differential equation which describes the use and recharging of charged tRNAs T is

$$\frac{dT}{dt} = \frac{V(\bar{T} - T(t))}{K_m + \bar{T} - T(t)} - \sum_{L'} k\rho_i(1-\rho_{i+1}),$$

where the sum is over all L' codons which require an aa-tRNA for elongation. We use no subscript on V, T, K_m since we consider here a mono-codon mRNA (a single type of tRNA). At steady state ($dT/dt = 0$) we identify the term under the sum as the current, and since $k = rT(t)$ we can write

$$k = r\bar{T} - \frac{rL'K_m J}{V - L'J},$$

i.e., k is a function of J. Inserting this into the above equations for the current and then solving for J gives equations for the current and density in each phase.

In the LD phase nearly all of the tRNAs are charged. Hence k is approximately constant $k \approx r\bar{T}$ and Eq. (1) holds. The system will enter the limited resources (LR) regime if the rate at which aa-tRNAs are used approaches the rate at which bare tRNAs are recharged. In the LD phase for low initiation rate we can assume that the ribosomes are well spaced, so that the rate of aa-tRNA use is approximately equal

*E-mail: c.a.brackley@abdn.ac.uk

to the number of particles on the lattice multiplied by the hopping rate. Using Eq. (1) and $k \approx r\bar{T}$ the aa-tRNA use rate is

$$\phi \approx L'\rho k \approx \frac{L'\alpha}{1 + (w-1)\alpha/(r\bar{T})}.$$

The maximum recharging rate is achieved when $T(t) = 0$, i.e., this rate is

$$\psi \approx \frac{V\bar{T}}{\bar{T} + K_m}.$$

Equating these two rates gives the value of α at which we expect to see the onset of LR

$$\alpha_{\mathrm{LR}} \approx \frac{V}{L'} \frac{r\bar{T}}{r(K_m + \bar{T}) - (w-1)\bar{T}^2 \frac{V}{L'}}. \quad (4)$$

References

[1] Chris A. Brackley, M. Camen Romano, Celso Grebogi, and Marco Thiel. Limited resources in a driven diffusion process. *Phys. Rev. Lett.*, 105:078102, 2010.

[2] Chris A. Brackley, M. Carmen Romano, and Marco Thiel. Slow sites in an exclusion process with limited resources. *Phys. Rev. E*, 82:051920, 2010.

[3] G. Lakatos and T. Chou. Totally asymmetric exclusion processes with particles of arbitrary size. *J. Phys. A*, 36:2027, 2003.

[4] Leah B. Shaw, R. K. P. Zia, and Kelvin H. Lee. Totally asymmetric exclusion process with extended objects: A model for protein synthesis. *Phys. Rev. E*, 68(2):021910, Aug 2003.
