# Supplementary material for: The Dynamics of Supply and Demand in mRNA Translation
Source: PLoS Comput Biol. 2011 Oct 13;7(10):e1002203. doi: 10.1371/journal.pcbi.1002203 (PMC3192816; doi:10.1371/journal.pcbi.1002203)
Supplement: Text S2 — Includes the key for the labelling of codon/tRNA species, the values for enzyme kinetic constants used, and the sequences and description of each mRNA sequence given in table 1. Also includes the full list of mRNA species used in the larger scale simulations. (PDF) [file pcbi.1002203.s002.pdf]

The Dynamics of Supply and Demand in mRNA Translation

Supporting Information Text S2: Details of tRNAs, mRNAs and Recharging Parameters.

Chris A. Brackley[1*], M. Carmen Romano[1,2], Marco Thiel[1]

1 *Institute for Complex Systems and Mathematical Biology, SUPA, University of Aberdeen, Aberdeen, AB24 3UE, UK*
2 *Institute of Medical Sciences, Foresterhill, University of Aberdeen, Aberdeen, AB25 2ZD, UK*

1 Which tRNAs and codons are which?

μ	**Codon(s)**	**tRNA species**	**Amino Acid**
1	GCU, GCC	Ala1	Alanine
2	GCA, GCG	Ala2	
3	CGG	Arg1	Arginine
4	CGU, CGC, CGA	Arg2	
5	AGA	Arg3	
6	AGG	Arg4	
7	AAU, AAC	Asn	Asparagine
8	GAU, GAC	Asp	Aspartic acid
9	UGU, UGC	Cys	Cysteine
10	CAA	Gln1	Glutamine
11	CAG	Gln2	
12	GAA	Glu3	Glutamic acid
13	GAG	Glu4	
14	GGU, GGC	Gly1	Glycine
15	GGA	Gly2	
16	GGG	Gly3	
17	CAU, CAC	His	Histidine
18	AUA	Ile1	Isoleucine
19	AUU, AUC	Ile2	
20	CUA, CUG	Leu1	Leucine
21	UUG	Leu3	
22	UUA	Leu4	
23	CUU, CUC	Leu5	
24	AAG	Lys1	Lysine
25	AAA	Lys2	
26	AUG	Met	Methionine
27	UUU, UUC	Phe	Phenylalanine
28	CCA, CCG	Pro1	Proline
29	CCU, CCC	Pro2	
30	UCU, UCC	Ser2	Serine
31	AGU, AGC	Ser3	
32	UCA	Ser4	
33	UCG	Ser5	
34	ACU, ACC	Thr1	Threonine
35	ACG	Thr2	
36	ACA	Thr3	
37	UGG	Trp	Tryptophan
38	UAU, UAC	Tyr	Tyrosine
39	GUU, GUC	Val1	Valine
40	GUA	Val2	
41	GUG	Valb2	

Table showing which codon and tRNA corresponds to each species label μ. Where multiple codons are listed for a single tRNA, these are all "read" by the same tRNA via wobble base pairing [3]. Throughout this work for simplicity we have assumed wobble codons are translated in exactly the same way (at the same rate) as other codons.

*E-mail: c.a.brackley@abdn.ac.uk

2 Recharging Enzyme Parameters

As detailed in the article, measured values for K_m and k_{cat} can be found for the literature for only a small number of synthetases. For this reason we use the average of the values given in the following table.

**Enzyme**	k_{cat} (s^{-1})	K_m (mol l^{-1})	**Reference**
Histidine tRNA synthetase	1.5	3.2×10^{-6}	[4]
Seryl tRNA synthetase (*S. cerevisiae*)	1.1	1.9×10^{-7}	[5]
Seryl tRNA synthetase (*E. coli*)	2.6	5.6×10^{-7}	[2]
Proline tRNA synthetase	0.26	4.2×10^{-6}	[6]
Arginine tRNA synthetase	26	2.5×10^{-6}	[7]

3 mRNAs A-D

Here we show the DNA nucleotide sequence and corresponding codon sequence using the μ labelling, for each of the example mRNAs A-D. Note that only the coding sequence is shown, i.e. the stop codon is not included since termination is not thought to be a limiting process.

3.1 mRNA A - YDR382W

This is a short, highly expressed (154 mRNAs per cell) ribosomal protein with the following coding sequence.

ATGAAATACTTAGCTGCTTACTTATTATTGGTTCAAGGTGGTAACGCTGCCCCATCCGCCGCTGAC
ATCAAGGCCGTCGTCGAATCTGTCGGTGCTGAAGTCGATGAAGCCAGAATCAACGAATTGTTGTC
CTCTTTGGAAGGTAAGGGCTCTTTGGAAGAAATCATCGCTGAAGGTCAAAAGAAGTTCGCTACTG
TTCCAACTGGTGGTGCTTCTTCTGCTGCTGCCGGTGCTGCCGGTGCTGCTGCCGGTGGTGATGCT
GCTGAAGAAGAAAAGGAAGAAGAAGCTAAGGAAGAATCTGATGATGACATGGGTTTTGGTTTATT
CGATTAA

In the codon labelling code defined in section 1 of the supplementary material the sequence is

26 25 38 22 1 1 38 22 22 21 39 10 14 14 7 1 1 28 30 1 1 8 19 24 1 39 39 12 30 39 14 1 12 39 8 12 1 5 19 7 12 21 21 30 30 21 12 14 24 14 30 21 12 12 19 19 1 12 14 10 24 24 27 1 34 39 28 34 14 14 1 30 30 1 1 1 14 1 1 14 1 1 1 14 1 4 8 1 1 12 12 12 24 12 12 12 1 24 12 12 30 8 8 8 26 14 27 14 22 27 8

3.2 mRNA B - YLR378C

Longer protein involved in protein secretion from the cell. On average 5 mRNAs per cell.

ATGTCCTCCAACCGTGTTCTAGACTTGTTTAAGCCATTTGAATCTTTCCTGCCGGAAGTGATTGCT
CCAGAAAGGAAGGTTCCATACAACCAGAAACTTATCTGGACAGGCGTTTCTCTACTGATCTTTTTG
ATTCTGGGCCAGATTCCGCTGTACGGGATCGTGTCCAGTGAGACTTCCGACCCTCTGTACTGGCTA
CGTGCCATGCTGGCCTCCAACCGTGGTACTTTACTGGAATTGGGTGTTTCGCCCATCATCACTTCA
TCTATGATTTTCCAATTTTTGCAGGGTACTCAGCTTTTACAAATCAGACCTGAGAGCAAGCAGGAC
AGAGAGCTGTTCCAAATTGCTCAAAAGGTGTGCGCTATTATTCTGATCTTGGGCCAAGCCCTTGTG
GTCGTCATGACAGGTAACTACGGTGCCCCTTCGGACCTCGGATTGCCCATCTGTTTGTTGTTAATC
TTTCAATTGATGTTTGCATCGCTGATTGTGATGTTATTAGACGAATTGCTATCTAAGGGTTACGGC
TTGGGTTCCGGTATTTCTCTGTTCACGGCAACCAATATTGCCGAACAAATTTTCTGGAGAGCGTTT
GCTCCTACTACAGTCAATTCCGGTCGTGGTAAGGAGTTCGAAGGTGCTGTGATTGCCTTTTTCCAT
CTTTTGGCTGTCAGAAAGGACAAGAAAAGAGCCCTTGTCGAGGCCTTTTACCGTACCAATCTACCT
AATATGTTCCAAGTGTTGATGACCGTGGCCATCTTCCTCTTTGTTTTATATTTACAAGGCTTCCGT
TACGAATTGCCCATCAGGTCAACCAAAGTGAGAGGTCAAATTGGTATCTACCCCATCAAACTCTTT
TATACTTCCAACACCCCAATCATGTTGCAGAGTGCATTGACTTCTAACATTTTCTTGATCTCTCAA

ATCCTTTTCCAGAAATACCCAACCAATCCATTGATTCGTTTGATCGGTGTTTGGGGTATCAGGCCG
GGCACCCAGGGCCCTCAAATGGCCTTGAGCGGGTTGGCCTACTACATCCAACCATTAATGTCTTTA
TCCGAAGCTCTTCTGGACCCTATCAAGACCATCGTCTACATCACATTTGTTCTTGGTTCATGCGCA
GTATTTTCCAAGACATGGATCGAAATCTCCGGCACTTCCCCACGTGACATTGCCAAACAATTCAAA
GATCAAGGCATGGTCATTAACGGTAAGAGAGAAACCTCCATTTACAGAGAATTGAAGAAGATCAT
TCCAACTGCTGCTGCTTTCGGCGGTGCTACCATCGGTGCTCTTTCTGTTGGCTCCGACCTACTAGG
TACTTTAGGTTCTGGGGCATCCATTTTGATGGCTACTACCACCATCTACGGCTACTACGAAGCTGC
CGCCAAGGAAGGTGGGTTTACTAAGAACCTCGTTCCAGGATTTTCTGATTTGATGTGA

26 30 30 7 4 39 20 8 21 27 24 28 27 12 30 27 20 28 12 41 19 1 28 12 6 24 39 28 38 7 11 25 23 19 37 36 14 39 30 20
20 19 27 21 19 20 14 11 19 28 20 38 16 19 41 30 31 13 34 30 8 29 20 38 37 20 4 1 26 20 1 30 7 4 14 34 22 20 12 21
14 39 33 29 19 19 34 32 30 26 19 27 10 27 21 11 14 34 11 23 22 10 19 5 29 13 31 24 11 8 5 13 20 27 10 19 1 10 24
41 9 1 19 19 20 19 21 14 10 1 23 41 39 39 26 36 14 7 38 14 1 29 33 8 23 15 21 29 19 9 21 21 22 19 27 10 21 26 27
2 33 20 19 41 26 22 22 8 12 21 20 30 24 14 38 14 21 14 30 14 19 30 20 27 35 2 34 7 19 1 12 10 19 27 37 5 2 27 1 2
9 34 36 39 7 30 14 4 14 24 13 27 12 14 1 41 19 1 27 27 17 23 21 1 39 5 24 8 24 25 5 1 23 39 13 1 27 38 4 34 7 20 2
9 7 26 27 10 41 21 26 34 41 1 19 27 23 27 39 22 38 22 10 14 27 4 38 12 21 29 19 6 32 34 25 41 5 14 10 19 14 19 38
29 19 25 23 27 38 34 30 7 34 28 19 26 21 11 31 2 21 34 30 7 19 27 21 19 30 10 19 23 27 11 25 38 28 34 7 28 21 19
4 21 19 14 39 37 14 19 6 28 14 34 11 14 29 10 26 1 21 31 16 21 1 38 38 19 10 28 22 26 30 22 30 12 1 23 20 8 29 1
9 24 34 19 39 38 19 36 27 39 23 14 32 9 2 40 27 30 24 36 37 19 12 19 30 14 34 30 28 4 8 19 1 25 10 27 25 8 10 14
26 39 19 7 14 24 5 12 34 30 19 38 5 12 21 24 24 19 19 28 34 1 1 1 27 14 14 1 34 19 14 1 23 30 39 14 30 8 20 20 14
34 22 14 30 16 2 30 19 21 26 1 34 34 34 19 38 14 38 38 12 1 1 1 24 12 14 16 27 34 24 7 23 39 28 15 27 30 8 21 26

3.3 mRNA C - YJL136C

A highly expressed (137 mRNAs per cell) ribosomal protein.

ATGGAAAACGATAAGGGTCAATTAGTTGAATTATATGTTCCAAGAAAGTGTTCTGCTACCAACAG
AATCATCAAAGCCGATGACCACGCTTCTGTTCAAATCAACGTTGCCAAGGTTGATGAAGAAGGCCG
CGCCATTCCAGGTGAATACATCACTTACGCTTTGTCCGGTTACGTTAGATCCAGAGGTGAATCCGA
TGACTCTTTGAACCGTTTGGCTCAAAACGATGGTTTGTTGAAGAACGTTTGGTCTTACTCCCGTTA
A

26 12 7 8 24 14 10 22 39 12 22 38 39 28 5 24 9 30 1 34 7 5 19 19 25 1 8 8 17 1 30 39 10 19 7 39 1 24 39 8 12 12 14
4 1 19 28 14 12 38 19 34 38 1 21 30 14 38 39 5 30 5 14 12 30 8 8 30 21 7 4 21 1 10 7 8 14 21 21 24 7 39 37 30 38 3
0 4

3.4 mRNA D - YMR307W

A long protein involved in cell biosynthesis. On average 63 copies per cell.

ATGTTGTTTAAATCCCTTTCAAAGTTAGCAACCGCTGCTGCTTTTTTTGCTGGCGTCGCAACTGCG
GACGATGTTCCAGCGATTGAAGTTGTTGGTAATAAGTTTTTCTACTCCAACAACGGTAGTCAGTTC
TACATAAGAGGTGTTGCTTATCAGGCTGATACCGCTAATGAAACTAGCGGATCTACTGTCAACGAT
CCTTTGGCCAATTATGAGAGTTGTTCCAGAGATATTCCATACCTCAAAAAATTGAACACAAATGTT
ATCCGTGTCTACGCTATCAATACCACTCTAGATCACTCCGAATGTATGAAGGCTTTGAATGATGCT
GACATCTATGTCATCGCTGATTTAGCAGCTCCAGCCACCTCTATCAATAGAGACGATCCAACTTGG
ACTGTTGACTTGTTCAACAGCTACAAAACCGTTGTTGACACTTTTGCTAATTACACCAACGTTTTG
GGTTTCTTCGCCGGTAATGAAGTTACTAACAATTACACCAACACAGATGCATCTGCTTTCGTGAAG
GCAGCTATTAGAGACGTCAGACAATACATCAGCGACAAGAACTACAGAAAAATTCCAGTTGGCTAC
TCTTCCAATGATGACGAAGATACCAGAGTTAAGATGACTGATTATTTCGCTTGTGGTGATGATGAT
GTTAAGGCTGATTTTTACGGTATTAATATGTATGAATGGTGTGGTAAATCTGACTTCAAAACTTCT
GGTTATGCTGATAGAACTGCAGAATTCAAAAACTTATCTATTCCTGTTTTCTTCTCTGAATACGGT
TGTAACGAAGTAACACCAAGACTATTTACTGAGGTTGAAGCCTTGTACGGTTCTAATATGACAGAT
GTCTGGTCTGGTGGTATCGTATACATGTACTTCGAAGAGACTAACAAATACGGTTTAGTTAGCATC
GATGGTAATGATGTTAAAACTTTGGATGACTTCAACAACTATTCTTCTGAAATCAACAAAATATCA

CCAACTTCCGCCAACACAAAGTCTTACAGTGCAACAACAAGTGATGTTGCTTGTCCAGCTACTGGT
AAGTACTGGTCCGCTGCAACAGAATTACCACCAACTCCAAACGGAGGCTTGTGTTCATGTATGAAT
GCAGCCAATAGTTGTGTTGTTTCCGATGACGTTGATTCTGATGATTACGAAACCTTATTTAACTGG
ATCTGTAATGAAGTCGACTGTAGCGGTATTTCAGCAAACGGTACCGCCGGTAAGTATGGTGCTTA
CTCTTTCTGTACACCAAAGGAACAGCTATCTTTCGTTATGAATTTGTACTACGAGAAGAGTGGTGG
TAGCAAATCTGACTGTAGCTTCAGCGGTTCTGCCACTCTACAAACTGCCACCACGCAAGCTAGTTG
CTCCTCCGCTTTGAAAGAGATTGGTAGTATGGGTACCAACTCTGCATCAGGTAGTGTTGATTTGG
GTTCCGGAACTGAATCCAGTACTGCCTCTTCTAACGCTTCGGGGTCTTCTTCCAAGTCTAACTCCG
GCTCTTCTGGTTCTTCCAGTTCTTCTTCTTCTTCAGCTTCATCTTCATCTTCTAGCAAGAAGAA
TGCTGCCACCAACGTTAAAGCTAACTTAGCACAAGTGGTCTTTACCTCCATCATTTCCTTATCCAT
TGCCGCTGGTGTCGGTTTTGCTTTGGTTTAA

26 21 27 25 30 23 32 24 22 2 34 1 1 1 27 27 1 14 39 2 34 2 8 8 39 28 2 19 12 39 39 14 7 24 27 27 38 30 7 7 14 31 1 1 27 38 18 5 14 39 1 38 11 1 8 34 1 7 12 34 31 15 30 34 39 7 8 29 21 1 7 38 13 31 9 30 5 8 19 28 38 23 25 25 21 7 36 7 39 19 4 39 38 1 19 7 34 34 20 8 17 30 12 9 26 24 1 21 7 8 1 8 19 38 39 19 1 8 22 2 1 28 1 34 30 19 7 5 8 8 28 34 37 34 39 8 21 27 7 31 38 25 34 39 39 8 34 27 1 7 38 34 7 39 21 14 27 27 1 14 7 12 39 34 7 7 38 34 7 36 8 2 30 1 27 41 24 2 1 19 5 8 39 5 10 38 19 31 8 24 7 38 5 25 19 28 39 14 38 30 30 7 8 8 12 8 34 5 39 24 26 34 8 38 27 1 9 1 4 8 8 8 39 24 1 8 27 38 14 19 7 26 38 12 37 9 14 25 30 8 27 25 34 30 14 38 1 8 5 34 2 12 27 25 7 22 30 19 29 39 27 27 30 12 38 14 9 7 12 40 36 28 5 20 27 34 13 39 12 1 21 38 14 30 7 26 36 8 39 37 30 14 14 19 40 38 26 38 27 12 1 3 34 7 25 38 14 22 39 31 19 8 14 7 8 39 25 34 21 8 8 27 7 7 38 30 30 12 19 7 25 18 32 28 34 30 1 7 36 24 30 38 31 2 36 36 31 8 39 1 9 28 1 34 14 24 38 37 30 1 2 36 12 22 28 28 34 28 7 15 14 21 9 32 9 26 7 2 1 7 31 9 39 39 30 8 8 39 8 30 8 8 38 12 34 22 27 7 37 19 9 7 12 39 8 9 31 14 19 32 2 7 14 34 1 14 24 38 14 1 38 30 27 9 36 28 24 12 11 2 0 30 27 39 26 7 21 38 38 13 24 31 14 14 31 25 30 8 9 31 27 31 14 30 1 34 20 10 34 1 34 35 10 1 31 9 30 30 1 21 25 13 19 14 31 26 14 34 7 30 2 32 14 31 39 8 21 14 30 15 34 12 30 31 34 1 30 30 7 1 33 16 30 30 30 24 30 7 30 14 30 30 14 30 30 31 30 30 30 30 30 32 1 32 30 32 30 30 31 24 24 7 1 1 34 7 39 25 1 7 22 2 10 41 39 27 34 30 19 19 30 2 2 30 19 1 1 14 39 14 27 1 21 39

4 mRNAs used in Larger Scale Simulations

Here we give details of all of the group I and group II mRNAs used in the large scale simulations. The labels correspond to the number given to each mRNA in Fig. 15 of the main paper. Abundance data is from [1].

4.1 Group I mRNAs

Label	Protien name	Protien function	Length (codons)	Abundance in G1 Simulation	Abundance in G2 Simulation
1	YAL040C	G1 cyclin	581	168	1
2	YGL003C	cyclin ubiquitination	567	168	1
3	YGL116W	cyclin ubiquitination	611	1	168
4	YGR108W	G2 cyclin	472	1	168
5	YGR109C	G1 cyclin	381	168	1
6	YLR079W	inhibitor in G1/S transition	285	168	1
7	YMR199W	G1 cyclin	547	168	1
8	YPL256C	G1 cyclin	546	168	1
9	YPR119W	activator in G2/M transition	492	1	168
10	YPR120C	G1 cyclin	436	168	1

4.2 Group II mRNAs

Label	Protien name	Protien function	Length codons	Abundance in Cell [1]	Abundance in Simulation
11	YKL097W-A	cell wall	93	410.98	168
12	YLR110C	cell wall	134	398.14	162
13	YPL079W	ribosomal	161	324.25	132
14	YHR174W	enzyme	438	284.78	116
15	YGR192C	lycase	333	271.74	111
16	YKL060C	enzyme	360	261.85	107
17	YDL130W	ribosomal	107	252.34	103
18	YLR044C	enzyme	564	232.95	95
19	YCR012W	kinase	417	228.46	93
20	YBR191W	ribosomal	161	227.00	92
21	YKR057W	ribosomal	88	219.44	89
22	YBR118W	elongation factor	459	195.19	79
23	YDR050C	enzyme	249	182.94	74
24	YPR080W	elongation factor	459	178.54	72
25	YGL031C	ribosomal	156	173.72	71
26	YMR116C	ribosomal	320	170.92	69
27	YIL018W	ribosomal	255	170.18	69
28	YFR031C-A	ribosomal	255	166.58	68
29	YBL072C	ribosomal	201	162.86	66
30	YDL229W	ATPase	614	161.02	65
31	YNL135C	isomerase	115	22.96	9
32	YER160C	non-ribosomal	1756	22.75	9
33	YNL030W	histone	104	22.73	9
34	YGR060W	enzyme	310	22.65	9
35	YKR059W	initiation factor	396	22.37	9
36	YJR104C	enzyme	155	22.23	9
37	YML073C	ribosomal	177	21.88	8
38	YHR214C-B	non-ribosomal	1794	21.48	8
39	YLR264W	ribosomal	68	21.47	8
40	YHR026W	enzyme	214	21.46	8
41	YER056C-A	ribosomal	122	21.17	8
42	YEL026W	RNA binding	127	20.26	8
43	YGR254W	enzyme	438	20.18	8
44	YOR234C	ribosomal	108	19.73	8
45	YNL301C	ribosomal	187	20.10	8
46	YHL033C	ribosomal	257	19.79	8
47	YLL050C	actin filaments	144	17.31	7
48	YLR355C	amino acid synthesis	396	19.57	8
49	YNL209W	helps in protein folding	614	18.96	7
50	YMR142C	ribosomal	200	18.72	7
51	YPR098C	membrane	162	2.85	1
52	YLL039C	ubiquitin	382	2.84	1
53	YMR309C	initiation factor	813	2.84	1
54	YDR432W	RNA binding	415	2.84	1
55	YML110C	ubiquitin one biosynthesis	308	2.84	1
56	YER154W	membrane	403	2.84	1
57	YLR285W	lifespan	262	2.84	1
58	YER044C	ERGosterol biosynthesis	149	2.84	1
59	YPL012W	ribosome export	1229	2.83	1
60	YPR148C	unknown	436	2.83	1
61	YOR206W	ribosome production	711	2.82	1
62	YNL231C	lipid control	352	2.82	1
63	YGL234W	enzyme	803	2.82	1
64	YPL050C	enzyme	396	2.81	1
65	YDR465C	enzyme	413	2.80	1
66	YMR298W	enzyme	151	2.80	1
67	YOR117W	enzyme	435	2.80	1
68	YPR086W	transcription factor	346	2.80	1
69	YMR238W	membrane	459	2.79	1
70	YJL121C	enzyme	239	2.79	1

References

[1] Andreas Beyer, Jens Hollunder, Heinz-Peter Nasheuer, and Thomas Wilhelm. Post-transcriptional Expression Regulation in the Yeast Saccharomyces cerevisiae on a Genomic Scale. *Mol Cell Proteomics*, 3(11):1083–1092, 2004.

[2] Franck Borel, Christine Vincent, Reuben Leberman, and Michael Hartlein. Seryl-trna synthetase from escherichia col: implication of its n-termspecificity. *Nucl. Acids Res.*, 22, 1994.

[3] F.H.C. Crick. Codon–anticodon pairing: The wobble hypothesis. *J. Mol. Biol.*, 19(2):548 – 555, 1966.

[4] Christopher Francklyn, Jodi Adamd, and John Augustine. Catalytic defect in mutants of class ii histidyl-trna synthetase from salmonella typhimurium previously linked to decreased control of histidine biosynthesis regulation. *J. Mol. Biol.*, 280:847–858, 1998.

[5] Boris Lenhard, Sanda Filipić, Irena Landeka, Ivan Škrtić, Dieter Söll, , and Ivana Weygand-urašević. Defining the active site of yeast seryl-trna synthetase. *J. Biol. Chem.*, 272:1136–1141, 1997.

[6] Hongjian Liu, Richard Peterson, Jason Kessler, and Karin Musier-Forsyth. Molecular recognition of trnapro by escherichia coli proline trna synthetase in vitro. *Nucl. Acids Res.*, 23:165–169, 1995.

[7] Qing-shuo Zhang, En-duo Wang, and Ying-lai Wang. The role of tryptophan residues in escherichia coli arginyl-trna synthetase. *Biochimica et Biophysica Acta*, 1387:136–142, 1998.
