# Supplementary material for: The Dynamics of Supply and Demand in mRNA Translation
Source: PLoS Comput Biol. 2011 Oct 13;7(10):e1002203. doi: 10.1371/journal.pcbi.1002203 (PMC3192816; doi:10.1371/journal.pcbi.1002203)
Supplement: Text S3 — Includes results for simulations of mRNA C and D individually. (PDF) [file pcbi.1002203.s003.pdf]

The Dynamics of Supply and Demand in mRNA Translation

Supporting Information Text S3: Individual Simulations for mRNAs C and D.

Chris A. Brackley[1*], M. Carmen Romano[1,2], Marco Thiel[1]

1 *Institute for Complex Systems and Mathematical Biology, SUPA, University of Aberdeen, Aberdeen, AB24 3UE, UK*
2 *Institute of Medical Sciences, Foresterhill, University of Aberdeen, Aberdeen, AB25 2ZD, UK*

Here we present simulation data for mRNAs C and D treated individually. Figures 1 and 2 show results analogous to those of mRNAs A and B. In each case we observe slightly different behaviour, but common to both, we note that the point of the onset of the μ−LR induced queueing is less well defined than in the previous cases.

For mRNA C we note from Fig. 1(f) that several of the tRNA types become depleted to some degree, but the queueing is not clearly visible. Whilst our analytical method correctly predicts which codon type will become slow, and we do see dips in the density profile immediately to the right of these codons ($\mu = 30$, coloured red), we also see this to some extent behind codons of type $\mu = 38$ (green).

The smoothness of the onset of queueing can be attributed to two factors. Firstly, the fact that several aa-tRNA types become depleted means that there is a spread of initiation rates at which these codons become rate limiting. Also the slow codons are very close to the initiation site, and edge effects always smooth a transition to queueing [1].

For mRNA D (Fig. 2), we see queues behind codons of type $\mu = 11$ as predicted by our estimate of α_c, but we also see less severe queues at other points (e.g. behind codons of type $\mu = 23$ at $i = 81$). This is seen both at low density (Fig. 2(c)) as well as during queueing (Fig. 2(d)), and we note the difference in the scales on the vertical axis in these two plots. The fact that our estimate for α_c does approximate the point of the onset of queueing indicates that $\mu = 11$ codons do control the maximum protein production rate. Figure 2(f) shows that $\mu = 23$ aa-tRNAs are also significantly depleted, again contributing to the smoothness of the transition to queueing.

References

[1] M. Carmen Romano, Marco Thiel, Ian Stansfield, and Celso Grebogi. Queueing phase transition: Theory of translation. *Phys. Rev. Lett.*, 102(19):198104, 2009.

*E-mail: c.a.brackley@abdn.ac.uk

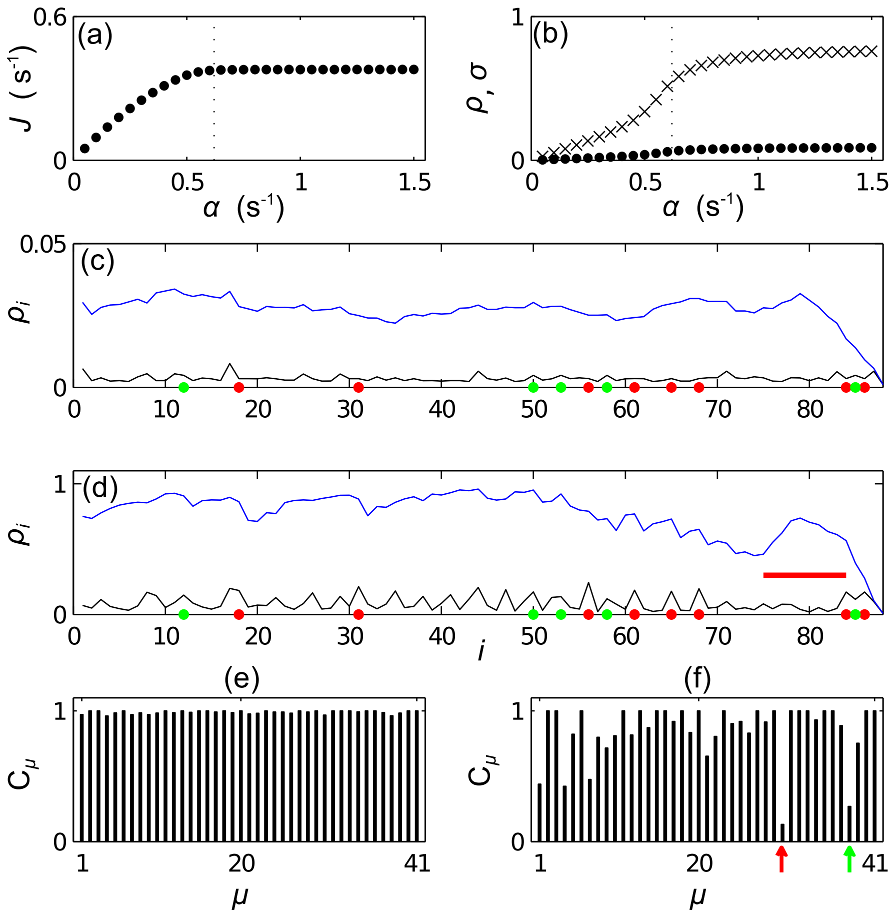

Figure 1: **Simulation results for mRNA C.** Plots (a) and (b) show how the current (protein production rate) and the mean density of ribosomes on the mRNA depend on the initiation rate α, respectively. In (b) the points show the reader density ρ and the crosses the coverage density σ. Plots (c) and (d) show ribosome density as a function of position i for small ($\alpha = 0.05\ \mathrm{s}^{-1}$) and large ($\alpha = 1.5\ \mathrm{s}^{-1}$) initiation rate respectively. Black lines show the reader density ρ_i and blue lines the coverage density σ_i. Red dots show the positions of codons of type $\mu = 30$, green dots codons of type $\mu = 38$, and the red bar indicates the width of the ribosomes. Bar graphs (e) and (f) show the steady state charging rate C_μ of each tRNA type. (e) shows $\alpha = 0.05\ \mathrm{s}^{-1}$ and (f) $\alpha = 1.5\ \mathrm{s}^{-1}$, the same values as in (c) and (d). The dotted lines in (a) and (b) are at α^c, which is where $\mu = 30$ (red) codons become slow.

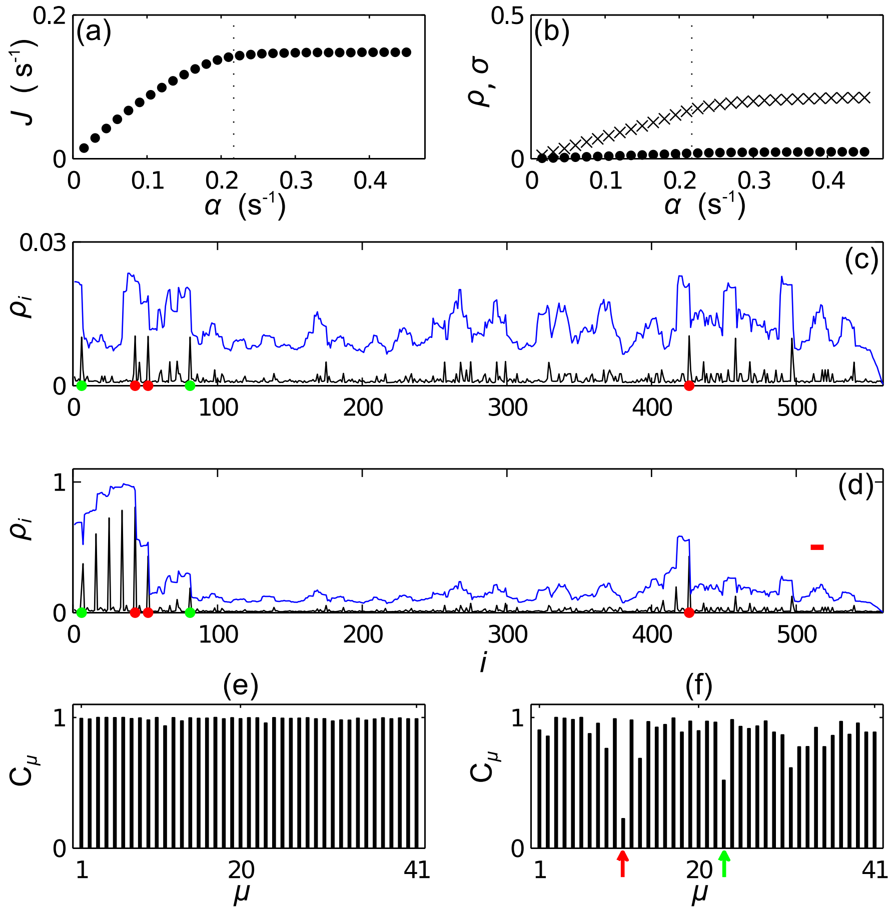

Figure 2: **Simulation results for mRNA D.** Plots are as in Fig. 1. Plots (c) and (e) are for $\alpha = 0.015$ s^{-1} and (d) and (f) for $\alpha = 0.45$ s^{-1}. The red dots show codons of type $\mu = 11$, and green dots codons of type $\mu = 23$. The dotted line is at α^c, which is where $\mu = 11$ (red) codons become slow.
