# Supplementary material for: The Dynamics of Supply and Demand in mRNA Translation
Source: PLoS Comput Biol. 2011 Oct 13;7(10):e1002203. doi: 10.1371/journal.pcbi.1002203 (PMC3192816; doi:10.1371/journal.pcbi.1002203)
Supplement: Text S4 — Includes further results for simulations of mixtures of mRNAs A and B, and mixtures of mRNAs C and D. (PDF) [file pcbi.1002203.s004.pdf]

The Dynamics of Supply and Demand in mRNA Translation

Supporting Information Text S4: Further Results for Mixtures of two mRNAs.

Chris A. Brackley[1*], M. Carmen Romano[1,2], Marco Thiel[1]

1 *Institute for Complex Systems and Mathematical Biology, SUPA, University of Aberdeen, Aberdeen, AB24 3UE, UK*
2 *Institute of Medical Sciences, Foresterhill, University of Aberdeen, Aberdeen, AB25 2ZD, UK*

Here we present further results for mixtures of two different mRNAs. For ease of comparison we reproduce some results which are shown in the main article. Discussion of the results is given in the main article.

*E-mail: c.a.brackley@abdn.ac.uk

Mixtures of mRNAs A and B

50:50 Mixture of mRNAs A and B by number of codons

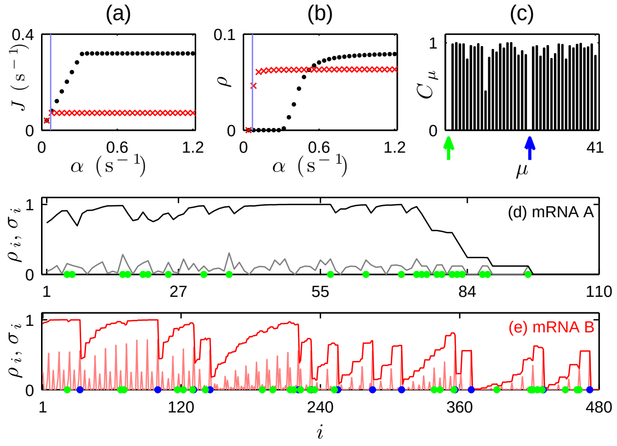

50:50 Mixture of mRNAs A and B by number of codons

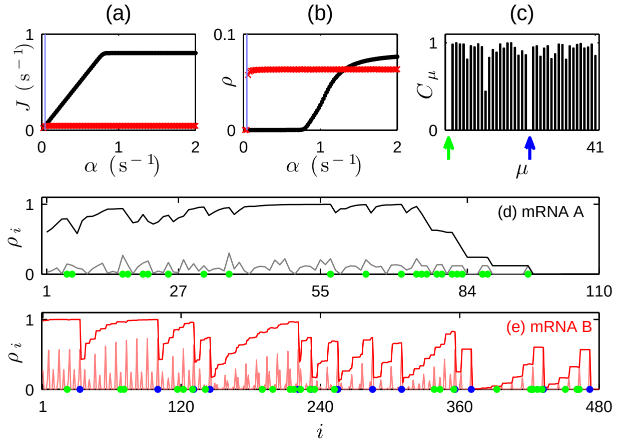

50:50 Mixture of mRNAs A and B by number of codons

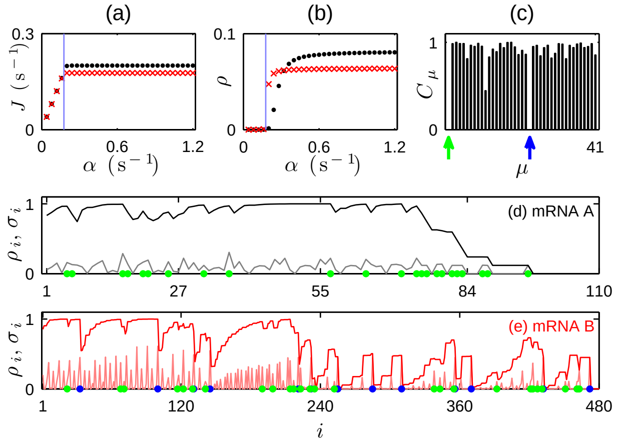

In each case (a) and (b) show J and ρ as a function of α for mRNAs of type A (black points) and type B (red crosses) (the same initiation rates are used for each species). The blue line shows α^c, where blue labelled codons cause queueing. Plot (c) shows the charging levels of tRNAs for large α ($\alpha = 1.2$ s^{-1} for the 50:50 and 80:20 cases, and $\alpha = 2$ s^{-1} for the 20:80 case). (d) and (e) show the site dependent reader (pale lines) and coverage (dark lines) density for each mRNA type, for the same value of α as in (c). The codons corresponding to the first aa-tRNA to become depleted are highlighted with blue dots ($\mu = 23$), and those for the second in green ($\mu = 1$).

Mixtures of mRNAs C and D

50:50 Mixture of mRNAs C and D by number of codons

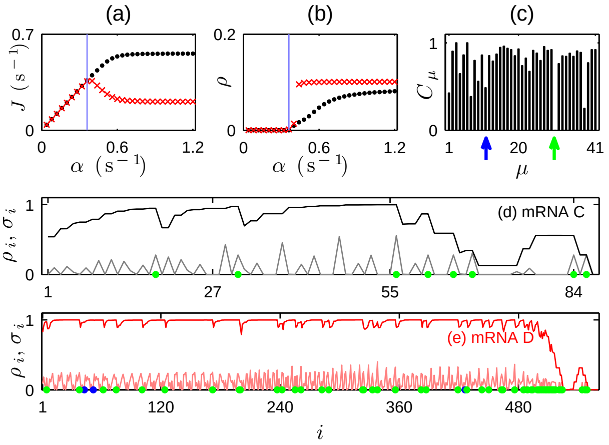

50:50 Mixture of mRNAs C and D by number of codons

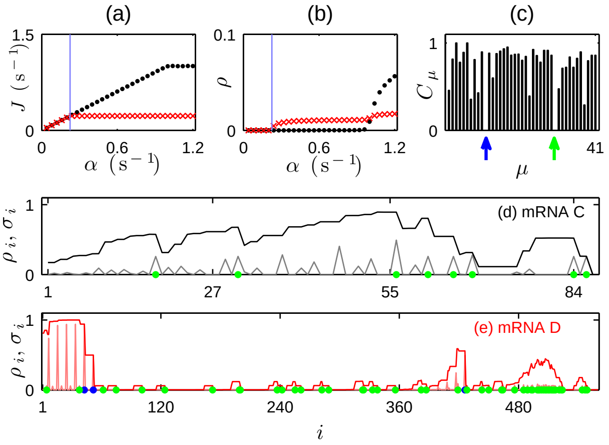

50:50 Mixture of mRNAs C and D by number of codons

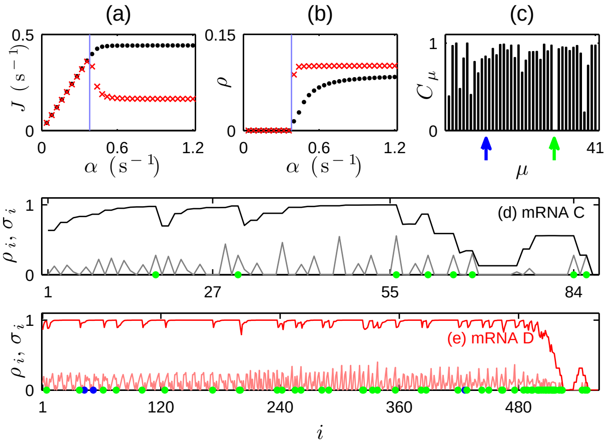

In each case (a) and (b) show J and ρ as a function of α for mRNAs of type A (black points) and type B (red crosses) (the same initiation rates are used for each species). The blue line shows α^c, where blue labelled codons cause queueing. Plot (c) shows the charging levels of tRNAs for $\alpha = 1.2$ s^{-1}. (d) and (e) show the site dependent reader (pale lines) and coverage (dark lines) density for each mRNA type, again for $\alpha = 1.2$ s^{-1}. The codons corresponding to the first aa-tRNA to become depleted are highlighted with blue dots ($\mu = 11$), and those for the second in green ($\mu = 30$).
