# Supplementary material for: The Dynamics of Supply and Demand in mRNA Translation
Source: PLoS Comput Biol. 2011 Oct 13;7(10):e1002203. doi: 10.1371/journal.pcbi.1002203 (PMC3192816; doi:10.1371/journal.pcbi.1002203)
Supplement: Text S5 — Comparison of the CAI and the QLI. (PDF) [file pcbi.1002203.s005.pdf]

The Dynamics of Supply and Demand in mRNA Translation

Supporting Information Text S5: Comparison with codon adaptation index.

Chris A. Brackley[1*], M. Carmen Romano[1,2], Marco Thiel[1]

1 *Institute for Complex Systems and Mathematical Biology, SUPA, University of Aberdeen, Aberdeen, AB24 3UE, UK*
2 *Institute of Medical Sciences, Foresterhill, University of Aberdeen, Aberdeen, AB25 2ZD, UK*

The codon adaptation index [1] is a measure of the translation efficiency of a given mRNA. Here we use our quantity Ω_μ (the likelihood that a given codon will become queue causing) to calculate a similar index, the queueing likelihood index (QLI) which we define

$$\mathrm{QLI} = \left(\prod_{i=1}^{L} \Omega_i \right)^{1/L}, \tag{1}$$

i.e., the geometric mean of the Ω_μ value for each codon on an mRNA. This gives an indication of the likelihood that queues will form on a given mRNA due to changes in supply or demand - i.e. it is also a measure of "translatability". We compare values of this index with the CAI for all mRNAs in the yeast genome. We note that the Pearson correlation coefficient for the two values is -0.808. This value is negative since a large value of the QLI indicates a high likelihood of queueing and therefore low translation efficiency.

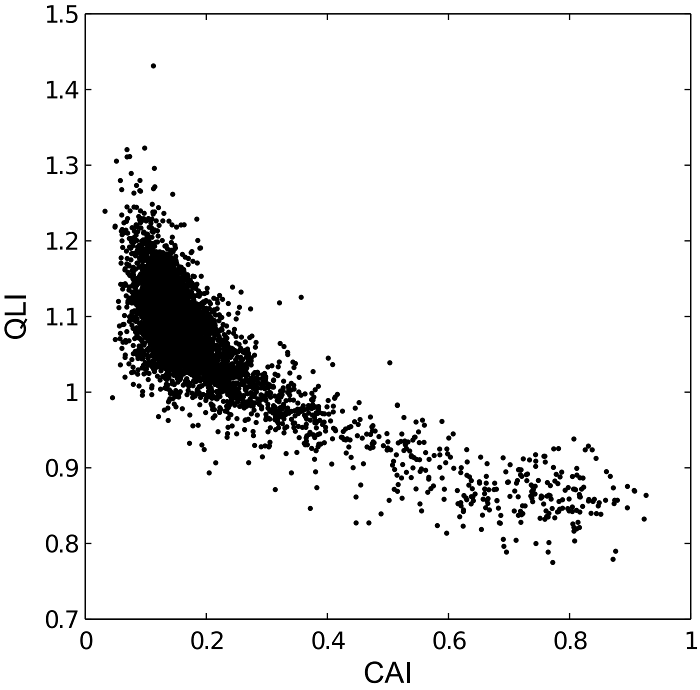

Figure 1: Plot comparing CAI (calculated as in [1]) and QLI for all mRNAs in the yeast genome. Abundances of mRNAs and tRNAs used to calculate Ω_μ values are from [2] and [3] respectively.

References

[1] Sharp P, Li W (1987) The codon adaptation index–a measure of directional synonymous codon usage bias, and its potential applications. Nucleic Acids Res 15: 1281-95.

[2] Beyer A, Hollunder J, Nasheuer HP, Wilhelm T (2004) Post-transcriptional Expression Regulation in the Yeast Saccharomyces cerevisiae on a Genomic Scale. Mol Cell Proteomics 3: 1083-1092.

[3] Percudani R, Pavesi A, Ottonello S (1997) Transfer rna gene redundancy and translational selection in saccharomyces cerevisiae. Journal of Molecular Biology 268: 322 - 330.
